# Does Adiponectin Inform Cardiovascular Risk in Older Adults? The ARIC Study

**DOI:** 10.1016/j.jacadv.2025.101625

**Published:** 2025-02-20

**Authors:** Layla A. Abushamat, Xiaoming Jia, Lu Xu, Chao Cheng, Chiadi E. Ndumele, Caroline Sun, B. Gwen Windham, Kunihiro Matsushita, Bing Yu, Vijay Nambi, Biykem Bozkurt, Jane E.B. Reusch, Casey M. Rebholz, Elizabeth Selvin, Christie M. Ballantyne, Ron C. Hoogeveen

**Affiliations:** a Section of Cardiovascular Research, Population Science, Department of Medicine, Baylor College of Medicine, Houston, Texas, USA; b Section of Cardiology, and Population Science, Department of Medicine, Baylor College of Medicine, Houston, Texas, USA; c Section of Epidemiology and Population Science, Department of Medicine, Baylor College of Medicine, Houston, Texas, USA; d Department of Epidemiology, Johns Hopkins Bloomberg School of Public Health, Baltimore, Maryland, USA; e Johns Hopkins Ciccarone Center for the Prevention of Cardiovascular Disease, Johns Hopkins University School of Medicine, Baltimore, Maryland, USA; f Department of Medicine, University of Mississippi Medical Center, Jackson, Mississippi, USA; g Department of Epidemiology, Human Genetics and Environmental Sciences, School of Public Health, University of Texas Health Science Center, Houston, Texas, USA; h Section of Cardiology, Michael E. DeBakey Veterans Affairs Medical Center, Houston, Texas, USA; i Winters Center for Heart Failure Research, Baylor College of Medicine, Houston, Texas, USA; j Department of Medicine, University of Colorado Anschutz Medical Campus, Aurora, Colorado, USA

**Keywords:** adiponectin, aging, cardiovascular disease, heart failure, NT-proBNP

## Abstract

**Background:**

Adiponectin, an atheroprotective adipokine, is associated with adverse outcomes in older age. It is unclear whether this is due to overlapping pathophysiological pathways with N-terminal pro–B-type natriuretic peptide (NT-proBNP).

**Objectives:**

The authors investigated adiponectin's associations with cardiovascular disease (CVD) risk in older adults.

**Methods:**

Among Atherosclerosis Risk in Communities prospective cohort study participants without baseline CVD at visit 5 (n = 4,729, mean age 75), adiponectin and adiponectin/NT-proBNP category associations with incident CVD events (heart failure [HF], atherosclerotic cardiovascular disease, and death during median follow-up of 5.5 years) and echocardiographic parameters were assessed. Metabolomic signatures of adiponectin/NT-proBNP categories were explored.

**Results:**

Higher adiponectin was associated with older age, female sex, and less obesity, diabetes, and hypertension but increased risk for incident HF (HR: 1.91 [95% CI: 1.49-2.44], per natural-log unit increase) and CVD death (HR: 1.67 [95% CI: 1.19-2.32]). Interaction of NT-proBNP with adiponectin was significant for HF (*P*-interaction = 0.03). There was no significant association between adiponectin and heart failure with preserved ejection fraction after adjusting for NT-proBNP. Elevations of both biomarkers (A+ [upper tertile]/N+ [≥125 pg/mL]) had higher risk (vs A+/N−; HF: HR 5.41 [95% CI: 2.72-10.78]; CVD death: HR 3.50 [95% CI: 1.48-8.24]). Compared with A+/N−, A−/N+ had increased risk for HF (HR 2.84 [95% CI: 1.41-5.72]) while A−/N− had no increased event risk. A+/N+'s metabolomic signature (88% similar to NT-proBNP's) showed acylcarnitine species consistent with incomplete beta-oxidation; top-associated metabolites were significantly associated with HF and CVD death.

**Conclusions:**

Elevated adiponectin and NT-proBNP in older adults are associated with increased risk for HF and CVD death beyond traditional risk factors.

Adiponectin, an adipokine primarily secreted by adipose tissue, is a marker of adipose tissue function and is inversely related to body weight, insulin resistance, and type 2 diabetes.^1^ Adiponectin is anti-inflammatory and antiatherogenic.^2^ Elevated adiponectin in individuals without coronary artery disease is associated with decreased future risk for myocardial infarction.^3^ However, in those with cardiovascular disease (CVD) or CVD risk factors, higher adiponectin is associated with increased CVD death and all-cause mortality.

This phenomenon extends to heart failure (HF), in which rising adiponectin is associated with disease severity and increased mortality.^4^ Higher adiponectin has been associated with higher natriuretic peptide levels and HF risk in older adults.^5^^,^^6^ Mendelian randomization analyses have shown adiponectin concentrations increase by 11.4% for every doubling of genetically determined N-terminal pro–B-type natriuretic peptide (NT-proBNP), despite no causal association of adiponectin with heart function.^7^ The association of adiponectin with adverse cardiovascular outcomes in older adults may reflect a common pathway with NT-proBNP.

Aging increases the burden of oxidative stress, endothelial dysfunction, and mitochondrial dysfunction, pathways also implicated in atherosclerotic cardiovascular disease (ASCVD) and HF.^8^^,^^9^ In older individuals, adiponectin is associated with physical deconditioning, reduced muscle mass and density, weight loss,^10^ and frailty,^11^ potentially representing a marker of metabolic derangement, namely oxidative stress and mitochondrial dysfunction, related to age-related wasting,^12^ all of which are linked to poor ASCVD and HF outcomes.

HF prevalence increases with age,^13^ and incident HF is the leading CVD event in older adults.^14^ Given the weaker associations of traditional CVD risk factors with CVD outcomes at older age,^15^ particularly for HF,^16^ identifying other influential factors may be useful for HF risk stratification in older populations. We hypothesized that NT-proBNP impacts the association of adiponectin with HF. We used data from the ARIC (Atherosclerosis Risk In Communities study to assess the relationship between adiponectin and CVD events, including HF, in older adults across phenotypes categorized by adiponectin/NT-proBNP levels. We explored the metabolic signature of these categories to identify pathways relevant to the interplay among adiponectin, NT-proBNP, and HF.

## Methods

### Study population

ARIC^17^ is a prospective observational cohort study of CVD incidence in adults recruited from 4 U.S. communities. ARIC visit 5 (2011-2013) was the index visit for this analysis.

### Biomarkers

Adiponectin and NT-proBNP were measured in EDTA plasma (collected at visit 5; stored at −70 °C) using immunoturbidimetric assay (Denka Seiken) and electrochemiluminescent immunoassay (Roche), respectively. Cardiac structure and function were assessed by comprehensive 2-dimensional, Doppler, tissue Doppler, and speckle-tracking echocardiography performed at visit 5.^18^ A predefined imaging protocol with uniform imaging hardware and software was used for echocardiographic acquisition and processing.

Metabolomic profiling was performed on visit 5 serum samples (stored at −80 °C) by Metabolon Inc using untargeted gas chromatography/mass spectrometry– and liquid chromatography/mass spectrometry–based quantification.^19^

### Outcomes

Primary outcomes were incident HF hospitalization, coronary heart disease (CHD), stroke, CVD mortality, and all-cause mortality. Incident HF hospitalization was ascertained by expert panel adjudication; HF was further adjudicated as heart failure with preserved ejection fraction (HFpEF) (left ventricular ejection fraction [LVEF] ≥50%) or heart failure with reduced ejection fraction (HFrEF) (LVEF <50%).^20^ Incident CHD events encompassed fatal CHD and definite or probable myocardial infarction.^21^ Incident stroke events included ischemic strokes and definite or probable hospitalized embolic or thrombotic strokes based on diagnosis codes as well as hospital records and neuroimaging reports.^22^ CVD deaths were ascertained from hospital discharge records and death certificates. The cutoff date for administrative censoring for events was December 31, 2017, for the primary analysis and December 31, 2020, for metabolic profiling.

As an exploratory outcome, frailty at visit 5 was assessed using measures of grip strength, walking speed, weight loss, energy, and physical activity. Individuals were classified as frail (>3 components present), prefrail (1 or 2 components present), or robust (0 components present).^23^

### Statistical analysis

Adiponectin was evaluated in categorical (tertiles) and continuous (natural log–transformed values) analyses. Associations between adiponectin at visit 5 and incident HF hospitalization, CHD, stroke, and CVD death after visit 5 were assessed by Cox proportional hazard models. Model 1 adjusted for age, sex, and race. Model 2 additionally adjusted for total cholesterol, high-density lipoprotein cholesterol (HDL-C), current smoking, systolic blood pressure (SBP), antihypertensive medication use, diabetes status, body mass index (BMI), estimated glomerular filtration rate (eGFR), lipid-lowering medication use, and high-sensitivity C-reactive protein (hs-CRP). Model 3 additionally adjusted for NT-proBNP levels. Interaction was tested for sex, race, diabetes status, BMI, and NT-proBNP; stratified analysis was performed for variables with significant interaction. Because of a high death rate after visit 5, we performed sensitivity analysis evaluating risk for incident CVD events competing with nonevent death. To assess the proportional hazards assumption of the model, graphical examination of log(−log(survival)) vs log(survival time) was used to confirm the curves were roughly parallel. Shapes of associations between adiponectin and outcomes were evaluated with adiponectin levels modeled as restricted cubic splines. Cross-sectional associations of adiponectin with NT-proBNP and echocardiographic parameters were investigated using linear regression analysis adjusted as above.

The relationship of adiponectin and NT-proBNP with CVD events was further evaluated by categorizing participants by both adiponectin and NT-proBNP levels: elevated adiponectin (upper tertile) with elevated NT-proBNP (≥125 pg/mL) (A+/N+), nonelevated adiponectin (lower 2 tertiles) with elevated NT-proBNP (A−/N+), elevated adiponectin with nonelevated NT-proBNP (<125 pg/mL) (A+/N−), and nonelevated adiponectin with nonelevated NT-proBNP (A−/N−). Risk for outcome events was compared across categories using Cox regression models described above. The A+/N− subgroup was used as reference because it had the lowest cardiometabolic risk profile (lower rates of hypertension, diabetes, and elevated BMI). In exploratory analysis, we assessed the association of adiponectin/NT-proBNP categories with robustness and frailty at visit 5, using multinomial logistic regression adjusted by model 1.

For metabolomic analysis, logistic regression adjusting for model 2 was used to identify metabolites associated with adiponectin and NT-proBNP. Multivariable logistic regression including model 2 variables was performed to identify metabolites with significantly different levels between A+/N+ and A+/N− subgroups. To correct for multiple testing, the Benjamini-Hochberg method was used to obtain the false discovery rate (FDR). We performed least absolute shrinkage and selection operator logistic regression to identify metabolites that can distinguish A+/N+ vs A+/N− samples. The R package “glmnet” was used for the analysis. Top metabolites of each subgroup were assessed for risk for outcome events using Cox regression analysis with model 2 adjustment.

Stata version 16 (StataCorp), SAS version 9.4 (SAS Institute Inc), and R version 3.3.3 were used for the statistical analyses. Orange was used for data visualization of metabolomics results.^24^ Pathway analyses were performed using MetaboAnalyst 5.0.^25^

See Supplemental Material for additional details.

## Results

Of 6,538 participants at visit 5, we excluded individuals with prevalent CHD (n = 1,013), stroke (n = 174), or HF (n = 444), race other than White or Black (n = 18) and Black participants at Minneapolis or Washington field centers (n = 24) because of small numbers, and participants missing adiponectin data (n = 136). After exclusions, 4,729 participants were included in the primary analysis; metabolomic data were available for 4,006 (Supplemental Figure 1).

### Baseline characteristics

Participants with higher adiponectin levels were more likely to be older, women, and White and to have lower BMI, waist-to-hip ratio, fasting glucose, diastolic blood pressure, eGFR, and triglycerides, and higher total cholesterol, HDL-C, and low-density lipoprotein cholesterol levels (Table 1). They were also less likely to have diabetes or hypertension or to be on antihypertensive or lipid-lowering medications. Females had higher median levels of adiponectin and NT-proBNP (Supplemental Table 1). Associations of adiponectin with clinical characteristics adjusted for age, sex, and race can be found in Supplemental Table 2.

**Table 1:** Baseline Characteristics Across Adiponectin Tertiles

	Overall (n = 4,729)	Adiponectin Tertiles (μg/mL)			*P* Trend
		1 (0.2-7.8)	2 (7.9-13.4)	3 (13.5-50.0)	
Age, y	75.3 ± 5.13	74.4 ± 4.79	75.3 ± 4.94	76.3 ± 5.45	<0.001
Female, %	62.53	45.05	62.90	79.87	<0.001
Black, %	22.65	36.42	19.15	12.20	<0.001
SBP, mm Hg	130.4 ± 17.68	130.5 ± 17.36	130.3 ± 17.35	130.5 ± 18.33	0.617
DBP, mm Hg	66.9 ± 10.39	68.0 ± 10.03	66.8 ± 10.29	66.0 ± 10.74	<0.001
Heart rate, beats/min	65.6 ± 10.93	65.4 ± 11.20	65.3 ± 10.68	66.1 ± 10.88	0.040
Antihypertensive medication use, %	70.07	78.72	70.01	61.36	<0.001
Hypertension, %	71.14	78.20	70.78	64.34	<0.001
Lipid-lowering medication use, %	50.27	58.26	51.62	40.77	<0.001
Current smoking, %	5.80	5.70	5.46	6.26	0.519
Diabetes, %	28.91	43.32	26.92	16.06	<0.001
BMI, kg/m^2^	28.5 ± 5.59	30.4 ± 5.43	28.8 ± 5.22	26.3 ± 5.33	<0.001
Waist/hip ratio	0.93 ± 0.080	0.96 ± 0.067	0.93 ± 0.076	0.90 ± 0.083	<0.001
eGFR, mL/min/1.73 m^2^	71.0 ± 16.47	71.9 ± 16.92	70.8 ± 16.35	70.4 ± 16.09	0.025
Triglycerides, mg/dL	111 (84, 149)	129 (96, 174)	114 (86, 151)	96 (76, 122)	<0.001
Total cholesterol, mg/dL	186.7 ± 40.86	174.9 ± 38.93	187.8 ± 40.08	197.7 ± 40.33	<0.001
HDL-C, mg/dL	53.4 ± 14.02	45.7 ± 10.45	52.6 ± 11.96	62.1 ± 14.27	<0.001
LDL-C (mg/dL)	108.4 ± 33.86	100.5 ± 32.95	109.9 ± 33.55	114.8 ± 33.50	<0.001
Fasting glucose, mg/dL	111.9 ± 26.88	120.6 ± 33.26	110.7 ± 22.33	104.4 ± 20.51	<0.001
NT-proBNP, pg/mL	116.2 (61.3, 222.8)	80.5 (42.7, 147.3)	111.0 (60.3, 211.1)	171.4 (97.9, 314.7)	<0.001
hs-CRP, mg/L	1.99 (0.95, 4.12)	2.42 (1.20, 4.95)	2.01 (0.96, 4.21)	1.58 (0.77, 3.29)	<0.001
Ejection fraction, %	65.9 ± 5.77	65.5 ± 5.65	66.2 ± 5.77	65.9 ± 5.86	0.060
LVMI, g/m^2^	74.5 (65.0, 86.7)	76.1 (65.4, 89.1)	74.5 (65.4, 85.7)	72.8 (64.0, 84.8)	<0.001
LAVI, mL/m^2^	24.0 (19.7, 29.1)	23.5 (19.3, 28.3)	24.0 (19.8, 29.0)	24.8 (20.1, 30.2)	<0.001
e′, cm/sec	6.8 (5.6, 8.2)	6.7 (5.6, 8.1)	6.8 (5.6, 8.1)	6.9 (5.7, 8.5)	0.002
E/e′, cm/s	9.4 (7.5, 11.8)	9.2 (7.5, 11.6)	9.4 (7.5, 11.9)	9.4 (7.6, 11.9)	0.034
Peak tricuspid regurgitation velocity, cm/s	235 (217, 254)	236 (219, 254)	235 (217, 253)	236 (217, 254)	0.803
Average peak longitudinal strain, %	18.4 (19.8, 16.7)	17.8 (19.4, 16.2)	18.6 (20.0, 17.0)	18.6 (20.1, 16.9)	<0.001

Values are mean ± SD, median (25th, 75th percentiles), or %. *P* values for linear trend were calculated by using nonparametric test of trend (an extension of Wilcoxon rank-sum test) for ranks across ordered groups.

BMI = body mass index; DBP = diastolic blood pressure; e′ = early diastolic mitral annular velocity; E/e′ = transmitral early peak velocity to early diastolic mitral annular velocity; eGFR = estimated glomerular filtration rate; HDL-C = high-density lipoprotein cholesterol; hs-CRP = high-sensitivity C-reactive protein; LAVI = left atrial volume index; LDL-C = low-density lipoprotein cholesterol; LVMI = left ventricular mass index; NT-proBNP = N-terminal pro–B-type natriuretic peptide; SBP = systolic blood pressure.

Median NT-proBNP levels were significantly higher in higher adiponectin tertiles. Adiponectin, under natural-log transformation, was positively associated with NT-proBNP (measured in pg/mL), under natural-log transformation (β = 0.16, *P* < 0.001) after adjustment for model 2 covariables (age, sex, race, total cholesterol, HDL-C, current smoking, SBP, antihypertensive medication use, diabetes status, BMI, eGFR, lipid-lowering medication use, and hs-CRP).

### Adiponectin and echocardiographic parameters

In adjusted linear regression models, adiponectin (natural log-transformed) was positively associated with left ventricular mass index , left atrial volume index, early diastolic mitral annular velocity, and peak tricuspid regurgitation velocity, and negatively associated with LVEF (Table 2).

**Table 2:** Association of Adiponectin and Echocardiographic Parameters

	Model	Beta Coefficient	95% CI	*P* Value
LVEF^a^	1	0.006	0.009-0.003	<0.001
	2	0.005	0.007-0.002	0.001
LVMI^b^	1	0.070	0.144-0.004	0.066
	2	0.121	0.054-0.189	<0.001
LAVI^b^	1	0.179	0.125-0.233	<0.001
	2	0.227	0.178-0.275	<0.001
e′^b^	1	0.257	0.199-0.315	<0.001
	2	0.126	0.074-0.178	<0.001
E/e′^b^	1	0.131	0.180-0.081	<0.001
	2	0.005	0.050-0.040	0.830
Peak TR velocity^b^	1	0.082	0.267-0.103	0.384
	2	0.223	0.054-0.391	0.010
Average peak longitudinal strain^c^	1	0.017	0.024-0.010	<0.001
	2	0.003	0.010-0.003	0.329

Linear regression analysis; adiponectin natural log transformed. Model 1 is adjusted by age, sex, and race; model 2 adjusted by model 1 covariables plus total cholesterol, HDL-C, current smoking, SBP, antihypertensive medication use, diabetes status, BMI, eGFR, lipid-lowering medication use, and log–hs-CRP.

BMI = body mass index; e′ = early diastolic mitral annular velocity; E/e′ = transmitral early peak velocity to early diastolic mitral annular velocity; eGFR = estimated glomerular filtration rate; HDL-C = high-density lipoprotein cholesterol; hs-CRP = high-sensitivity C-reactive protein; LAVI = left atrial volume index; LVEF = left ventricular ejection fraction; LVMI = left ventricular mass index; SBP = systolic blood pressure; TR = tricuspid regurgitation.

a Per % increase.

b Per natural log unit increase.

c Per unit increase.

### Adiponectin and CVD events

Over a median follow-up of 5.5 years (5.09, 6.03), there were 325 incident HF hospitalization events (in 6.9% of the study population), 178 incident CHD events (3.8%), 115 incident stroke events (2.4%), and 180 CVD deaths (3.8%). When adjusting for traditional risk factors (age, sex, race/center, total cholesterol, HDL-C, current smoking, SBP, antihypertensive medication use, diabetes status, BMI, eGFR, lipid-lowering medication use, and log–hs-CRP; model 2), higher adiponectin level was significantly associated with increased risk for incident HF hospitalization (HR: 1.91; 95% CI: 1.49-2.44; *P* < 0.001 per natural-log unit increase in adiponectin), CVD death (HR: 1.67; 95% CI: 1.19-2.32; *P* = 0.003), and all-cause mortality (HR: 1.69; 95% CI: 1.41-2.04; *P* < 0.001) (Table 3). Adiponectin was significantly associated with risk for HFpEF (HR: 1.98; 95% CI: 1.47-2.67; *P* < 0.001), but not HFrEF (HR: 1.41; 95% CI: 0.98-2.02; *P* = 0.063). In sensitivity analyses, association of adiponectin with HF hospitalization and HFpEF remained significant when competing with nonevent death (HR: 1.81; 95% CI: 1.39-2.36; *P* < 0.001) (Supplemental Table 3). Associations of adiponectin with HF hospitalization, HFpEF, and CVD death became nonsignificant when the model included NT-proBNP adjustment (Table 3).

**Table 3:** Association of Adiponectin and Incident CVD

Incident Event (n/N, %)	Incident Rate in 1,000 Person-Y (95% CI)	Model	HR	95% CI	*P* Value
CHD (178/4,729, 3.76%)	7.19 (6.21-8.33)	1	0.84	0.65-1.09	0.195
		2	1.19	0.86-1.64	0.283
		3	1.03	0.74-1.43	0.854
Ischemic stroke (115/4,729, 2.43%)	4.61 (3.84-5.54)	1	0.67	0.50-0.91	0.010
		2	0.88	0.60-1.29	0.513
		3	0.75	0.52-1.08	0.125
HF hospitalization (325/4,729, 6.87%)	13.20 (11.84-14.72)	1	1.35	1.11-1.65	0.003
		2	1.91	1.49-2.44	<0.001
		3	1.14	0.89-1.46	0.288
HFpEF (220/4,729, 4.65%)	8.87 (7.77-10.13)	1	1.43	1.12-1.82	0.004
		2	1.98	1.47-2.67	<0.001
		3	1.35	1.00-1.83	0.054
HFrEF (131/4,729, 2.77%)	5.25 (4.42-6.23)	1	1.14	0.84-1.54	0.411
		2	1.41	0.98-2.02	0.063
		3	0.75	0.54-1.05	0.091
CVD death (180/4,729, 3.81%)	7.16 (6.18-8.28)	1	1.36	1.05-1.77	0.022
		2	1.67	1.19-2.32	0.003
		3	1.00	0.73-1.39	0.978
Total mortality (578/4,729, 12.2%)	22.98 (21.18-24.93)	1	1.41	1.21-1.63	<0.001
		2	1.69	1.41-2.04	<0.001
		3	1.25	1.04-1.52	0.019

Adiponectin (natural log transformed) analyzed as a continuous variable. Model 1 adjusted by age, sex, and race/center; model 2 adjusted by model 1 covariables plus total cholesterol, HDL-C, current smoking, SBP, antihypertensive medication use, diabetes status, BMI, eGFR, lipid-lowering medication use, and log–hs-CRP. Model 3 adjusted by model 2 covariables plus log–NT-proBNP.

BMI = body mass index; CHD = coronary heart disease; CVD = cardiovascular disease; eGFR = estimated glomerular filtration rate; HDL-C = high-density lipoprotein cholesterol; HF = heart failure; HFpEF = HF with preserved ejection fraction; HFrEF = HF with reduced ejection fraction; hs-CRP = high-sensitivity C-reactive protein; n/N = Number of events/total participants at risk; NT-proBNP= N-terminal pro–B-type natriuretic peptide; SBP = systolic blood pressure.

**Figure 1:**
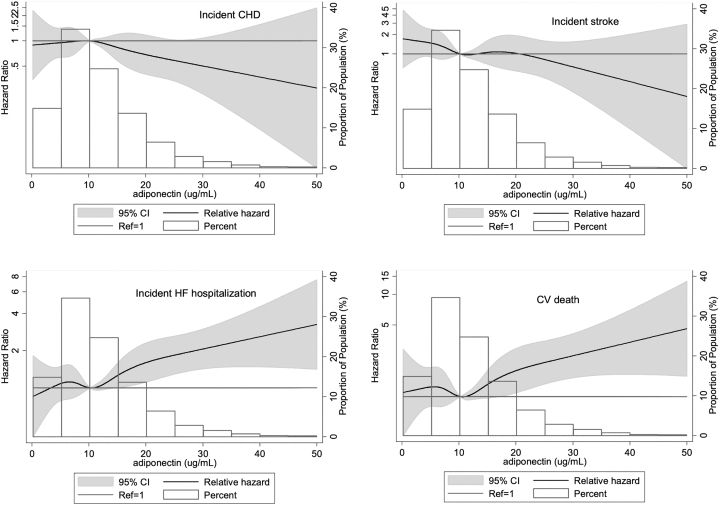
Restricted Cubic Splines Showing Associations of Adiponectin With Incident Events From 2011-2017 The median value (10.2 μg/mL) was used as reference in a Cox proportional hazard model adjusted for age, sex, and race. Knots were placed at the 5th, 27.5th, 50th, 72.5th, and 95th percentiles. CHD = coronary heart disease; CV = cardiovascular; HF = heart failure.

When adiponectin was analyzed categorically, the highest adiponectin tertile was associated with significantly greater risk for incident HF hospitalization (HR: 2.40; 95% CI: 1.71-3.38), HFpEF (HR: 2.33; 95% CI: 1.55-3.52), and CVD death (HR: 2.08; 95% CI: 1.32-3.29) compared with the lowest tertile after model 2 adjustment (Supplemental Table 4). When adiponectin was modeled as restricted cubic splines to assess the shapes of the associations between adiponectin and CVD events, the risk for incident HF hospitalization and CVD death appeared to increase monotonically without evidence of a threshold effect (Figure 1).

**Figure 2:**
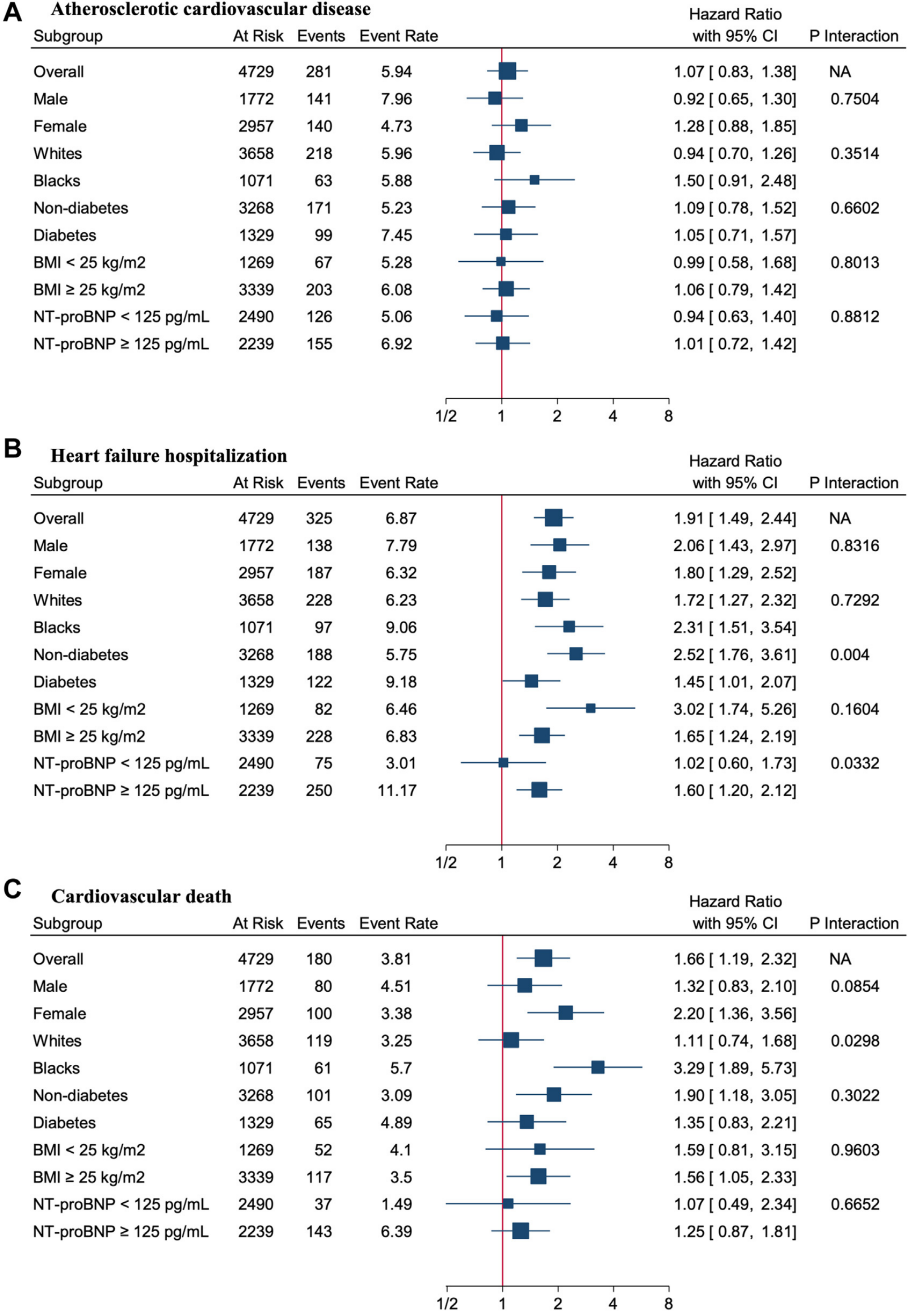
Association of Adiponectin with Cardiovascular Outcomes in the Overall Population and By Subgroup Associations of adiponectin with (A) incident atherosclerotic cardiovascular disease (composite of coronary heart disease and stroke), (B) heart failure hospitalization, and (C) cardiovascular death by subgroups. BMI = body mass index; NA = not applicable; NT-proBNP = N-terminal pro–B-type natriuretic peptide.

Interaction of diabetes status with adiponectin was significant for incident HF hospitalization, with stronger associations among participants without diabetes than with diabetes (HR: 2.52 [95% CI: 1.76-3.61] vs HR: 1.45 [95% CI: 1.01-2.07] after model 2 adjustment, *P*-interaction = 0.004). Interaction of NT-proBNP with adiponectin was significant for incident HF hospitalization; the association between adiponectin and HF events was significant only among participants with elevated NT-proBNP (HR: 1.60 [95% CI: 1.20-2.12] vs HR: 1.02 [95% CI: 0.60-1.73], *P*-interaction = 0.03). No significant interaction was noted by sex or BMI subgroups (Figure 2).

### Adiponectin, NT-proBNP, and CVD risk

**Figure 3:**
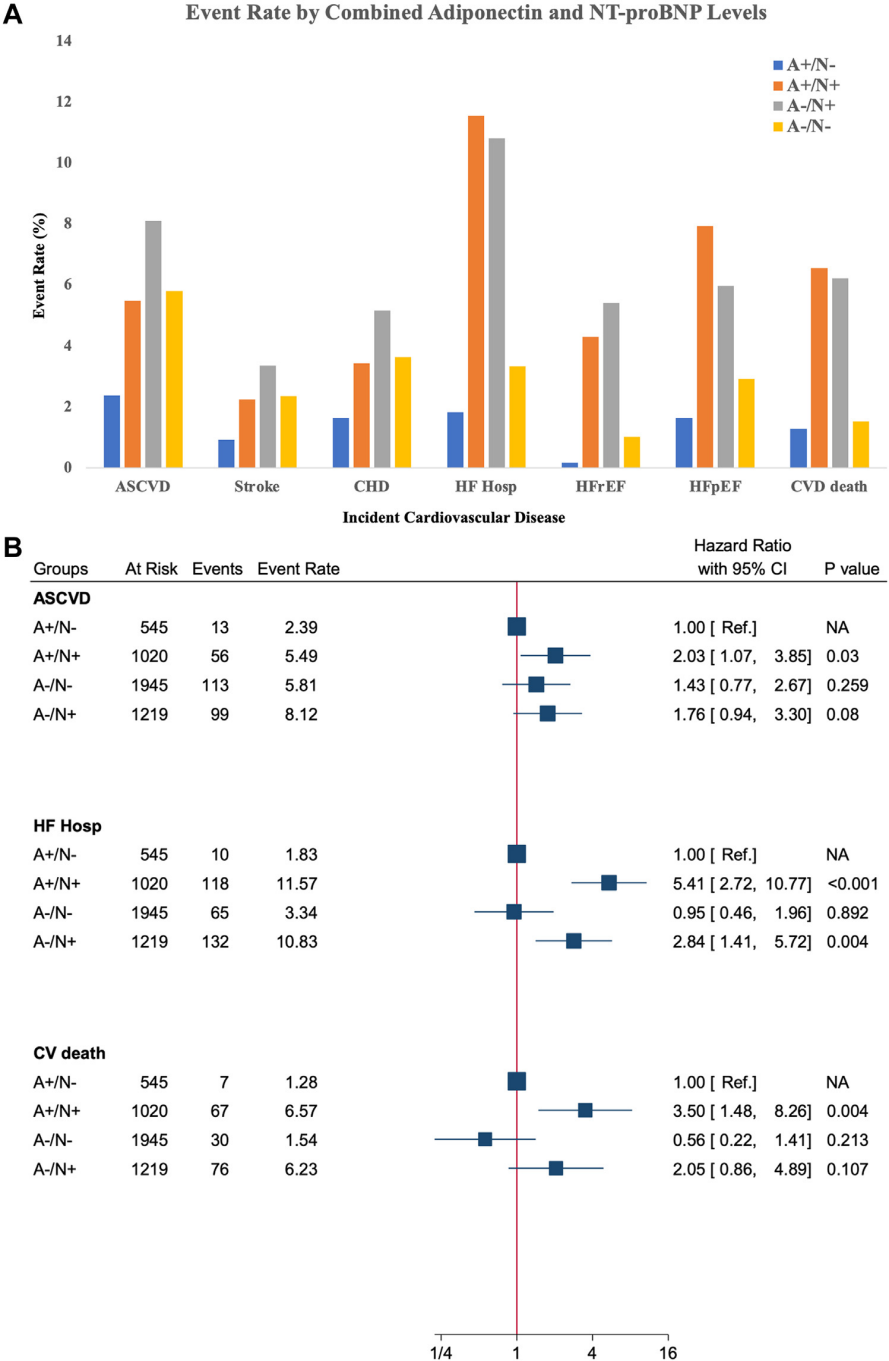
Incident Events by Combined Adiponectin and N-Terminal Pro–B-Type Natriuretic Peptide Levels (A) Absolute event rates; (B) HRs adjusted for age, sex, race, total cholesterol, high-density lipoprotein cholesterol, current smoking, systolic blood pressure, antihypertensive medication use, diabetes status, body mass index, estimated glomerular filtration rate, Lipid-lowering medication use, and log–high-sensitivity C-reactive protein. ASCVD = atherosclerotic cardiovascular disease; CHD = coronary heart disease; CV = cardiovascular; CVD = cardiovascular disease; HF= heart failure; HFpEF = heart failure with preserved ejection fraction; HFrEF = heart failure with reduced ejection fraction; NA = not applicable; NT-proBNP = N-terminal pro–B-type natriuretic peptide.

Adiponectin was significantly correlated with NT-proBNP (Spearman's correlation 0.366). Although many individuals had elevations of NT-proBNP and adiponectin, a substantial number of individuals had high levels of adiponectin with levels of NT-proBNP <125 pg/mL (normal per current guidelines^26^) (Supplemental Figure 2). Incorporating both adiponectin and NT-proBNP, we characterized 4 subgroups based on biomarker cut points as defined above: A+/N+ (n = 1,020), A−/N+ (n = 1,219), A+/N− (n = 545), and A−/N− (n = 1,945). Before adjustments for risk factors, the A+/N− subgroup had the lowest absolute risk for ASCVD, HF, and CVD death, whereas the A+/N+ subgroup appeared to have the highest risk for HF hospitalization and CVD death (Figures 3A and 3B; Supplemental Table 5A). We used the A+/N− subgroup as reference and found that the A+/N+ and A−/N+ subgroups had incrementally increasing risk for HF hospitalization (HR: 2.84 [95% CI: 1.41-5.72] and HR: 5.41 [95% CI: 2.72-10.78], respectively) and CVD death (HR: 2.05 [95% CI: 0.86-4.89] and HR: 3.50 [95% CI: 1.48-8.24], respectively) in model 2 adjustment. Compared with A+/N−, the A−/N− subgroup had similar risk for HF hospitalization (HR: 0.95 [95% CI: 0.46-1.96]) and CVD death (HR: 0.56 [95% CI: 0.22-1.40]), although CVD death rate was numerically lower. No significant differences were observed among subgroups with respect to CHD or stroke events; when combined as the composite ASCVD, the A+/N+ subgroup had significantly higher risk than the A+/N− subgroup (HR: 2.03 [95% CI: 1.07-3.84]) (Figure 3B; Supplemental Tables 5A to 5C).

Overall, the A+/N− subgroup appeared to have the most optimal cardiometabolic profile. Demographic, clinical, and anthropomorphic characteristics demonstrated that individuals in the A+/N+ subgroup tended to be older. The A+/N+ subgroup had similar SBP to that in the A−/N+ subgroup, which was higher than in the subgroups without elevated NT-proBNP. However, the A+/N+ subgroup was less likely to have hypertension or diabetes or to use antihypertensive or lipid-lowering medication, had lower triglycerides, fasting glucose, hs-CRP, BMI, and waist-to-hip ratio, and had higher HDL-C than the A−/N+ and A−/N− subgroups. The subgroups with elevated NT-proBNP (A+/N+ and A−/N+) had higher transmitral early peak velocity to early diastolic mitral annular velocity, left atrial volume index, and peak tricuspid regurgitation velocity compared with those without elevated NT-proBNP. Additionally, elevated NT-proBNP subgroups had modestly lower LVEF and longitudinal strain and higher left ventricular mass index than their counterparts without elevated NT-proBNP (Table 4).

**Table 4:** Baseline Characteristics Across Adiponectin and NT-proBNP Categories

	Adiponectin <13.5 μg/mL, NT-proBNP <125 pg/mL	Adiponectin <13.5 μg/mL, NT-proBNP ≥125 pg/mL	Adiponectin ≥13.5 μg/mL, NT-proBNP <125 pg/mL	Adiponectin ≥13.5 μg/mL, NT-proBNP ≥125 pg/mL	*P* Value
Age, y	73.8 ± 4.55	76.5 ± 4.96	74.4 ± 4.78	77.3 ± 5.54	<0.001
Female, %	49.61	60.87	80.55	79.51	<0.001
Black, %	32.49	20.34	13.39	11.57	<0.001
SBP, mm Hg	128.5 ± 16.25	133.5 ± 18.58	126.8 ± 16.84	132.5 ± 18.79	<0.001
DBP, mm Hg	68.1 ± 9.76	66.3 ± 10.71	65.8 ± 10.17	66.1 ± 11.03	<0.001
Antihypertensive medication use, %	71.55	78.90	53.03	65.82	<0.001
Hypertension, %	72.39	77.90	55.37	69.15	<0.001
Lipid-lowering medication use, %	53.91	56.61	40.77	40.77	<0.001
Current smoking, %	5.25	6.12	4.04	7.47	0.028
Diabetes, %	34.94	35.56	18.03	15.00	<0.001
eGFR, mL/min/1.73 m^2^	74.5 ± 15.36	66.2 ± 17.35	75.0 ± 14.56	67.9 ± 16.33	<0.001
Triglycerides, mg/dL	118 (89, 160)	125 (94, 167)	94 (75, 122)	97 (77, 122)	<0.001
Total cholesterol, mg/dL	181.7 ± 39.14	180.7 ± 41.41	204.6 ± 42.07	194.0 ± 38.88	<0.001
HDL-C, mg/dL	49.2 ± 11.77	49.0 ± 11.70	63.4 ± 14.38	61.4 ± 14.16	<0.001
LDL-C, mg/dL	106.0 ± 33.04	104.0 ± 34.41	120.7 ± 35.62	111.6 ± 31.89	<0.001
Fasting glucose, mg/dL	116.1 ± 27.28	114.9 ± 31.03	106.1 ± 22.41	103.4 ± 19.36	<0.001
NT-proBNP, pg/mL	58.4 (36.3, 84.8)	214.6 (158.4, 325.3)	80.1 (57.2, 100.2)	250.9 (176.7, 432.6)	<0.001
Adiponectin, μg/mL	7.4 (4.9, 9.8)	8.8 (6.5, 10.9)	17.3 (14.9, 20.6)	18.8 (15.7, 24.0)	<0.001
hs-CRP, mg/L	2.08 (1.02, 4.22)	2.46 (1.14, 5.31)	1.38 (0.72, 2.90)	1.73 (0.83, 3.42)	<0.001
hs-TnI, ng/L	2.6 (1.8, 4.0)	3.6 (2.4, 5.8)	2.3 (1.7, 3.2)	3.4 (2.3, 5.5)	<0.001
hs-TnT, ng/L	9 (6, 13)	11 (8, 17)	8 (6, 12)	10 (7, 16)	<0.001
BMI, kg/m^2^	29.8 ± 5.32	29.2 ± 5.47	26.3 ± 4.89	26.3 ± 5.56	<0.001
Waist/hip ratio	0.95 ± 0.073	0.94 ± 0.074	0.89 ± 0.080	0.90 ± 0.084	<0.001
Weight, kg	83.9 ± 16.55	79.8 ± 16.47	69.5 ± 14.17	69.2 ± 15.76	<0.001
Body fat, %	35.8 ± 8.82	35.9 ± 14.84	34.5 ± 8.30	33.0 ± 8.92	<0.001
Fat mass, kg	30.6 ± 11.18	28.9 ± 10.96	24.6 ± 9.53	23.6 ± 10.31	<0.001
Lean mass, kg	53.4 ± 11.04	50.9 ± 10.52	45.0 ± 8.52	45.7 ± 9.29	<0.001
Frailty, %					<0.001
Frail	3.70	5.94	4.22	8.24	
Prefrail	38.94	48.84	44.77	51.57	
Robust	57.36	45.21	51.01	40.20	
Ejection fraction, %	66.1 ± 5.17	65.5 ± 6.51	66.8 ± 5.29	65.3 ± 6.09	<0.001
LVMI, g/m^2^	73.3 (63.8, 84.8)	78.9 (68.3, 91.5)	71.2 (62.1, 79.6)	74.3 (65.0, 88.3)	<0.001
LAVI, mL/m^2^	22.4 (18.6, 26.7)	26.3 (21.7, 31.4)	22.0 (18.4, 27.0)	26.1 (21.4, 32.0)	<0.001
e′, cm/s	6.8 (5.7, 8.2)	6.7 (5.4, 8.0)	7.0 (5.7, 8.7)	6.9 (5.7, 8.4)	<0.001
E/e′, cm/s	9.0 (7.3, 11.2)	10.0 (7.9, 12.5)	9.2 (7.4, 11.2)	9.6 (7.7, 12.2)	<0.001
Peak TR velocity, cm/sec	231 (215, 250)	240 (222, 259)	227 (211, 245)	239 (222, 259)	<0.001
Average peak longitudinal strain, %	−18.3 (−19.6, −16.7)	−18.2 (−19.8, −16.4)	−18.9 (−20.2, −17.5)	−18.4 (−20.0, −16.6)	<0.001

Values are mean ± SD, %, or median (25th, 75th percentiles). *P* values were calculated by using one-way ANOVA, chi-square test, or Kruskal-Wallis rank test.

BMI = body mass index; DBP = diastolic blood pressure; e′ = early diastolic mitral annular velocity; E/e′ = transmitral early peak velocity to early diastolic mitral annular velocity; eGFR = estimated glomerular filtration rate; HDL-C = high-density lipoprotein cholesterol; hs-CRP = high-sensitivity C-reactive protein; hs-TnI = high-sensitivity troponin I; hs-TnT = high-sensitivity troponin T; LAVI = left atrial volume index; LDL-C = low-density lipoprotein cholesterol; LVMI = left ventricular mass index; NT-proBNP = N-terminal pro–B-type natriuretic peptide; SBP = systolic blood pressure; TR = tricuspid regurgitation.

The A+/N+ subgroup had the highest rate of frailty and lowest rate of robustness among the subgroups (Table 4). In multinomial logistic regression using A+/N− as the reference, the A+/N+ subgroup was significantly less likely to be robust vs frail (OR: 0.54 [95% CI: 0.33-0.89]); no significant difference was observed for the A−/N− (OR: 1.25 [95% CI: 0.75-2.07]) and A−/N+ (OR: 0.76 [95% CI: 0.46-1.25]) subgroups. Odds of being frail vs prefrail were not significantly different among subgroups (Supplemental Table 6).

### Adiponectin and A/N subgroups metabolomic signature and CVD events

**Figure 4:**
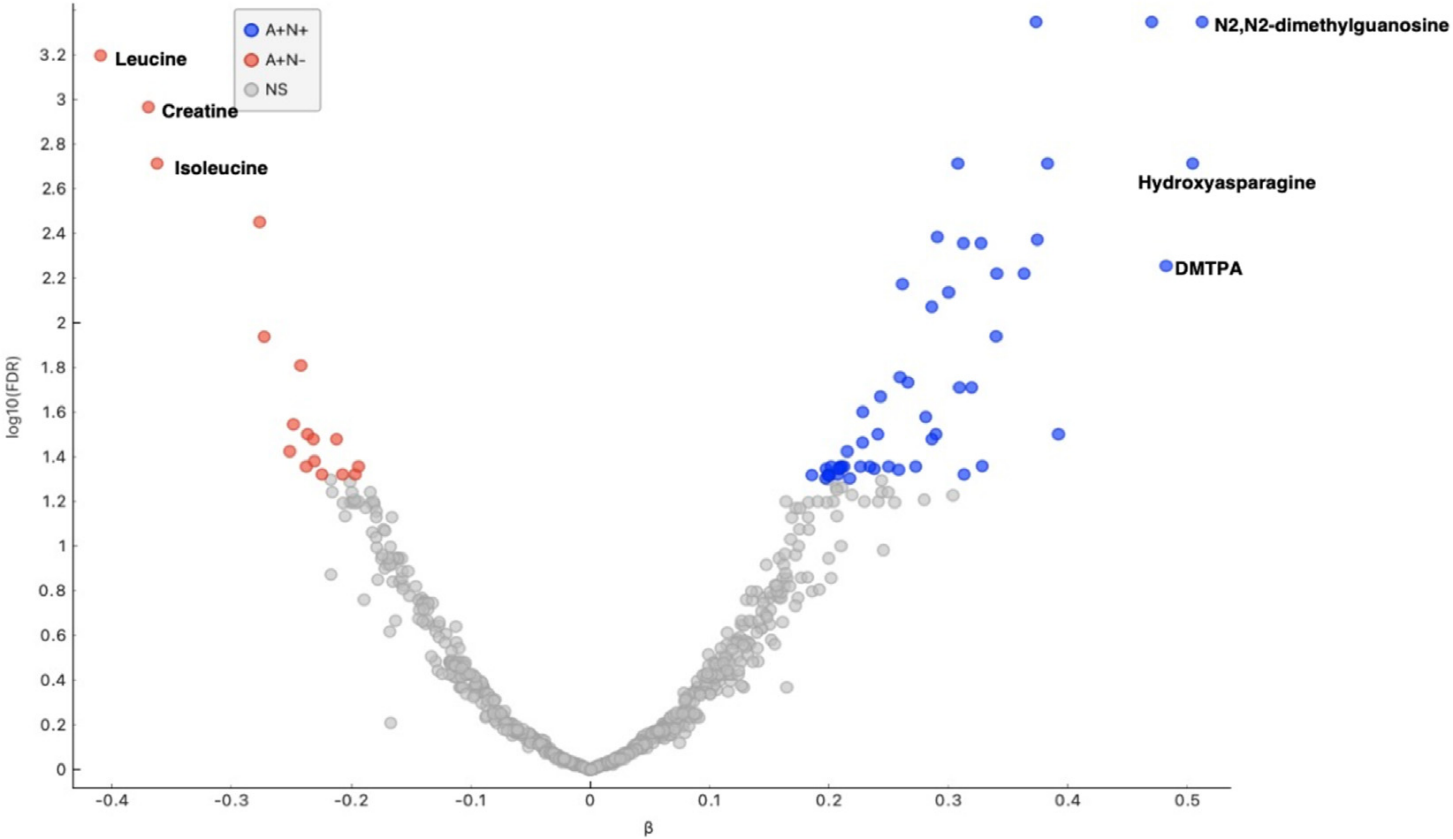
Metabolites Associated With Adiponectin/N-Terminal Pro–B-Type Natriuretic Peptide Subgroups Volcano plot depicting metabolites significantly associated with elevated adiponectin and N-terminal pro–B-type natriuretic peptide (A+/N+) and elevated adiponectin without elevated N-terminal pro–B-type natriuretic peptide (A+/N−) by effect size and significance. Top 3 Significant metabolites for each group are labeled. DMTPA = 2,3-dihydroxy-5-methylthio-4-pentenoate; FDR = false discovery rate; NS = not significant.

Using logistic regression analysis adjusting for covariates from model 2 above, 498/790 metabolites (63%) were significantly associated with adiponectin (FDR <0.05). Similarly, 332/790 (42%) were significantly associated with NT-proBNP. When assessing metabolite differential between A+/N+ and A+/N−, 71 metabolites were significantly associated with either subgroup (FDR <0.05) (Supplemental Table 7, Figure 4). The top correlated metabolite with A+/N+ was N2,N2-dimethylguanosine (β = 0.51, FDR <0.001). The acylcarnitine species C12 (β = 0.21, FDR = 0.05) and C20 (β = 0.20, FDR = 0.05) were among the 52 metabolites significantly associated with A+/N+. No acylcarnitine species were significantly associated with A+/N−. The top significant metabolic pathway associated with A+/N+ was the citrate cycle (Supplemental Table 8). Of note, 88% of metabolites significantly associated with A+/N+ were significantly associated with NT-proBNP. The top 3 metabolites associated with A+/N+ were N2,N2-dimethylguanosine, hydroxyasparagine, and 2,3-dihydroxy-5-methylthio-4-pentenoate. These were all significantly associated with incident HF hospitalization, HFpEF, CVD death, and total mortality. The top 3 metabolites associated with A+/N−, leucine, creatine, and isoleucine, were associated with decreased or no increased risk of HF hospitalization, CVD death, and total mortality (Supplemental Table 9).

## Discussion

In this study, we observed that higher adiponectin level in a cohort of older adults (mean age 75) without known clinical CVD was associated with increased risk for incident HF hospitalization and CVD death during ∼5.5-year follow-up. Higher adiponectin was also associated with structural findings of diastolic dysfunction and increased risk for HFpEF. However, this increased risk for HFpEF did not remain after adjusting for NT-proBNP.

Adiponectin was positively correlated with NT-proBNP, and NT-proBNP significantly interacted in the association between adiponectin and HF. The A+/N+ subgroup had higher CVD risk than other subgroups despite a better cardiometabolic profile (less diabetes, less hypertension, higher HDL-C, lower triglycerides, lower BMI, lower waist-to-hip ratio, lower body fat percentage) than those with lower adiponectin levels (Central Illustration). The A+/N− subgroup had lower risk for CVD events, were more robust, and had lower blood pressure with less antihypertensive use and better renal function than A+/N+. Additionally, top metabolites associated with A+/N+ had strong associations with HF hospitalization, CVD death, and total mortality, whereas those associated with A+/N− had a null or protective effect on these outcomes. However, 88% of significant metabolites associated with A+/N+ were significantly associated with NT-proBNP, suggesting NT-proBNP drives these relationships.

**Central Illustration:**
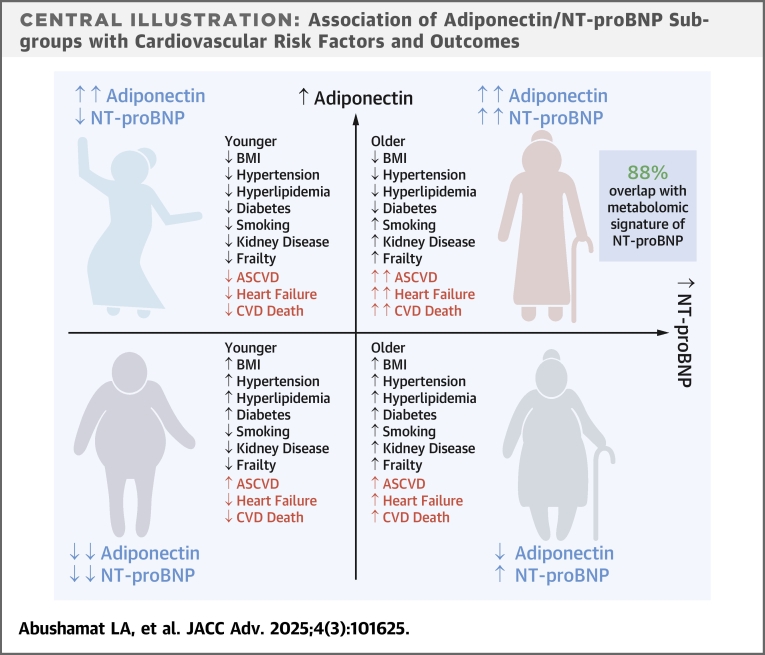
Association of Adiponectin/NT-proBNP Subgroups with Cardiovascular Risk Factors and Outcomes In older adults, concurrently elevated adiponectin and N-terminal pro–B-type natriuretic peptide identify a unique phenotype of increased cardiovascular risk despite an apparently favorable cardiometabolic profile. ASCVD = atherosclerotic cardiovascular disease; BMI = body mass index; CVD = cardiovascular disease; NT-proBNP = N-terminal pro–B-type natriuretic peptide. Created With BioRender.com.

NT-proBNP is a well-established biomarker of increased HF risk,^27^ and NT-proBNP ≥125 pg/mL is diagnostic of pre-HF (stage B HF) in current guidelines.^28^ We showed that in older adults, concurrently increased adiponectin and NT-proBNP further increased risk for HF hospitalization and CVD mortality. A case-cohort study showed similar findings of a significant association between adiponectin and total mortality only seen in the context of high NT-proBNP.^29^ Increased NT-proBNP in older age may be partially explained by elevated cardiac loading pressures and diastolic dysfunction but is also influenced by sex, race, and inflammation.^30^ Natriuretic peptides act on adipose tissue to induce lipolysis; interaction between adiponectin and natriuretic peptides has been speculated to drive the development of cardiac cachexia in patients with HF.^31^ As a key endocrine regulator of energy metabolism, adiponectin at elevated levels generally indicates a shift towards enhanced tissue energy utilization.^32^ In contrast, in patients with chronic HF or other chronic diseases, elevated adiponectin has been associated with catabolic wasting and worse outcomes.^4^ Our study suggests that the differential associations between adiponectin and cardiovascular outcomes may be related to NT-proBNP.

While higher adiponectin was positively associated with HF, CVD death, and total mortality when adjusting for age, sex, race, or traditional risk factors, we found this association is no longer significant when also adjusting for NT-proBNP in model 3 (Table 3). Additionally, with the A+/N+ subgroup having the highest event rate and A+/N- having the lowest event rate, our analysis suggests that the catabolic processes associated with elevated NT-proBNP appear to overcome any protective effects of adiponectin and drive poor outcomes, even in the setting of other favorable cardiovascular risk factors. These findings suggest that NT-proBNP may be an effect modifier or mediator in the association of adiponectin with HF in older age. Furthermore, the A+/N+ subgroup had lower hs-CRP levels than the A−/N+ subgroup, despite having the highest risk for incident HFpEF. HFpEF represents a substantial proportion of HF cases in older adults and is clinically a diverse syndrome with a range of phenotypic presentations,^33^ and the incidence of HFpEF increases with age.^34^ Our findings build upon previous data and show that in adults who have survived to older age free of clinical CVD, determinants of morbidity and mortality extend beyond traditional risk factors.^14^ The A+/N+ subgroup may represent a unique clinical phenotype of HFpEF in this older population.

Metabolomic analysis of the 2 high-adiponectin subgroups indicates underlying metabolic dysfunction in the A+/N+ subgroup. The association of acylcarnitine species with A+/N+ may indicate mitochondrial dysfunction; the even–chain acylcarnitine species noted in the metabolomic signature are derived from incomplete beta-oxidation of fatty acids.^35^ The top pathway associated with A+/N+ was the citrate cycle, consistent with potential defects in aerobic respiration. The high overlap of significant metabolites for A+/N+ with metabolites associated with NT-proBNP (including acylcarnitine species) is suggestive of a metabolically unfavorable environment highly influenced by NT-proBNP. The top 3 metabolites associated with the A+/N+ subgroup were all associated with increased incident CVD events, in contrast to the top 3 associated with A+/N−, which were neutral or associated with decreased incident CVD events. These data support the finding that NT-proBNP drives the association of elevated adiponectin with poor CVD outcomes.

In summary, we found that positive associations of adiponectin with HF and death in older adults previously reported in the literature are likely due to the effect of NT-proBNP. Clinically, concurrent elevation in adiponectin and NT-proBNP represents a phenotype of individuals with high HFpEF risk who may not be easily recognized by most clinicians based on an apparently favorable cardiometabolic profile. There has been a great deal of interest in understanding the heterogeneity of individuals who develop HFpEF. Although most HF studies routinely measure NT-proBNP, they do not measure adiponectin. Our finding that individuals with high levels of adiponectin have a different risk factor profile (less hypertension, diabetes, and obesity; older; and frail White women) and metabolomic profile supports the hypothesis that the pathogenesis may differ and thus the response to therapy may also differ. This should be examined in clinical trials and may be especially important in newer therapies such as glucagon-like peptide 1 or glucagon-like peptide-1/gastric inhibitory polypeptide, which lead to weight loss, including muscle mass loss. Future studies should also investigate the pathophysiological links between adiponectin and NT-proBNP.

Our study has several limitations. Our cohort comprised older adults without baseline clinical CVD; therefore, our results should be interpreted within this specific population and may not be readily extrapolated to individuals with pre-existing CVD. As this is an observational study, associations cannot be interpreted as causal. Adiponectin is influenced by sex, diabetes status, and adiposity (visceral vs subcutaneous adipose tissue), while NT-proBNP is affected by age and sex,^30^ race,^36^ and BMI (ie, lower in individuals with higher BMI),^37^ and cutoffs for categorical analysis were based on clinical thresholds^26^ rather than population-specific thresholds. The higher prevalence of HFpEF in older populations and the higher event rate for HFpEF compared to HFrEF in our study may introduce bias in associations identified with HFpEF. Lastly, metabolomic analysis was exploratory, limited to the 790 metabolites evaluated by Metabolon and limited by sample size; results were not validated in a separate cohort.

Our analysis also has several strengths. We leveraged circulating biomarker data, echocardiographic/Doppler parameters, and HF status to refine the relationship between adiponectin and risk for HF events. We elucidated the interaction of this relationship with NT-proBNP and sought to clarify these relationships by stratifying by NT-proBNP, assessing adiponectin's metabolomic signature, and comparing the A/N subgroup with highest risk for HF hospitalization and CVD death to the subgroup with the lowest CVD risk. We demonstrated that this association can be attributed to NT-proBNP and revealed a positive correlation between NT-proBNP and adiponectin. Furthermore, the metabolomic signature of the highest-risk group had significant overlap with that of NT-proBNP and reflected catabolic processes, including metabolic dysfunction and incomplete beta-oxidation. Overall, the extensive data available from the ARIC study, including metabolomic data, enabled a more comprehensive perspective on the association between adiponectin and cardiovascular health in older adults and facilitated the identification of a high-risk phenotype characterized by elevated adiponectin and NT-proBNP.

## Conclusions

Future cardiovascular morbidity and mortality in older adults without clinical CVD likely involve processes extending beyond traditional cardiac risk factors. We demonstrated that in this population, elevated adiponectin and NT-proBNP were positively associated with increased risk for HF hospitalization and CVD mortality despite a more favorable cardiometabolic profile, including less diabetes and obesity. This phenotype appears to be driven by similar pathophysiological processes found in the context of elevated NT-proBNP. Better understanding of these pathophysiological processes in this distinct phenotype could unveil strategies for CVD prevention in this high-risk group.

Perspectives **COMPETENCY IN MEDICAL KNOWLEDGE:** Adiponectin has been linked with increased CVD risk in older adults. NT-proBNP appears to interact with the association of adiponectin and CVD risk. Elevated adiponectin, in conjunction with elevated NT-proBNP, identifies a phenotype of older adults at high risk for clinical heart failure, especially heart failure with preserved ejection fraction, as well as CVD death, similar to those with low adiponectin and elevated NT-proBNP, despite having a better cardiometabolic profile compared with those with lower adiponectin levels. In contrast, individuals with elevated adiponectin without elevated NT-proBNP represent a more robust phenotype with lower CVD risk.

**TRANSLATIONAL OUTLOOK 1:** Adiponectin used in conjunction with NT-proBNP identifies older adults at higher risk for future heart failure events or CVD despite favorable cardiometabolic risk profiles, though this risk may be largely attributable to the effects of NT-proBNP.

**TRANSLATIONAL OUTLOOK 2:** Given the poor correlation of traditional risk factors with CVD events in older age, metabolomic analysis may identify other biomarkers, pathways, or signatures to improve risk prediction in this population.

## Funding support and author disclosures

The Atherosclerosis Risk in Communities study has been funded in whole or in part with Federal funds from the 10.13039/100000050National Heart, Lung, and Blood Institute, 10.13039/100000002National Institutes of Health, 10.13039/100000016Department of Health and Human Services, under Contract nos. (HHSN268201700001I HHSN268201700002I, HHSN268201700003I, HHSN268201700004I, HHSN268201700005I). Funding support for “Building on GWAS for NHLBI-diseases: the U.S. CHARGE consortium” was provided by the 10.13039/100000002NIH through the American Recovery and Reinvestment Act of 2009 (ARRA) (5RC2HL102419). Metabolomics measurements were sponsored by the 10.13039/100000051National Human Genome Research Institute (3U01HG004402-02S1). This work was supported by 10.13039/100000002NIH grant R01-HL134320 (ES, CMB); Dr Yu was in part supported by 10.13039/100000002NIH/10.13039/100000050NHLBI grant HL148218; Dr Rebholz was supported by 10.13039/100000002NIH/10.13039/100000050NHLBI grant R01-HL153178. Dr Selvin was supported by 10.13039/100000002NIH/10.13039/100000050NHLBI grant K24-HL152440 and 10.13039/100000002NIH/10.13039/100000062NIDDK grant R01-DK089174. Dr Abushamat was supported by Baylor College of Medicine 2022 Chao Physician-Scientist Award, an American Diabetes Association Cardiovascular Metabolism Award (#4-23-CVD1-01), and 10.13039/100000002NIH 10.13039/100000062NIDDK 1K23DK140641-01. Dr Matsushita received nonfinancial support from Roche Diagnostics outside of the submitted work. Dr Nambi is site principal investigator for studies funded by Amgen and Merck. Dr Bozkurt has had a consultation/advisory committee role for Abiomed, Amgen, Astra Zeneca, Boehringer Ingelheim, Cytokinetics, Daiichi Sankyo, Johnson & Johnson, Hanger Institute, Merck, Occlutech, Regeneron, Roche, Sanofi, sc Pharmaceuticals, Vifor, and Zoll/Respicardia; served on the clinical event committees of Abbott Vascular; and served on the data safety monitoring committees of Liva Nova, Cardurion, Novo Nordisk, and Renovacor. Dr Reusch receives payment (to her institution) from Medtronic and serves as an executive editor for Journal of Hypertension. Dr Selvin receives payments from Wolters Kluwer for chapters and laboratory monographs in UpToDate on measurements of glycemic control and screening tests for type 2 diabetes, in 2019; Dr Selvin received one honorarium from Novo Nordisk to speak at a scientific meeting on a topic of her choice. Dr Ballantyne has received grant/research support (to his institution) from Abbott Diagnostic, Akcea, Amgen, Arrowhead, Esperion, Ionis, Merck, New Amsterdam, Novartis, Novo Nordisk, Regeneron, and Roche Diagnostic and consultant fees from Abbott Diagnostics, Alnylam Pharmaceuticals, Althera, Amarin, Amgen, Arrowhead, Astra Zeneca, Denka Seiken, Esperion, Genentech, Gilead, Illumina, Ionis, Matinas BioPharma Inc, Merck, New Amsterdam, Novartis, Novo Nordisk, Pfizer, Regeneron, Roche Diagnostic, and TenSixteen Bio. Dr Hoogeveen has received grant/research support (to his institution) from Denka Seiken and is a consultant for Denka Seiken. All other authors have reported that they have no relationships relevant to the contents of this paper to disclose.
