# Supplementary material for: Does Adiponectin Inform Cardiovascular Risk in Older Adults? The ARIC Study
Source: JACC Adv. 2025 Feb 20;4(3):101625. doi: 10.1016/j.jacadv.2025.101625 (PMC11891717; doi:10.1016/j.jacadv.2025.101625)
Supplement: Supplemental Tables 1, 2, 3, 4, 5, 6, 7, 8, 9, and 10, and Supplemental Figures 1 and 2 [file mmc1.pdf]

**Supplemental Material**


| | | |
|---|---|---|
| **Supplemental Methods** | | 2–6 |
| **Supplemental Table 1:** | Median adiponectin and NT-proBNP levels by sex | 7 |
| **Supplemental Table 2:** | Associations of adiponectin (per in unit) with clinical characteristics, adjusted by age, sex, race. | 8 |
| **Supplemental Table 3:** | Association of adiponectin and incident CVD events: competing risk analysis with nonevent death | 9 |
| **Supplemental Table 4:** | Association of adiponectin tertile and incident CVD events | 10 |
| **Supplemental Table 5:** | Association of adiponectin and NT-proBNP categories with incident CVD. A. All subgroup analysis using A+/N- as Reference; B. A+/N+ vs A-/N+ (reference); C. A+/N- vs A-/N- (reference). | 11-12 |
| **Supplemental Table 6:** | Association between adiponectin and NT-proBNP categories and robustness/frailty score by multinomial logistic regression model | 13 |
| **Supplemental Table 7:** | Metabolites significantly associated with elevated adiponectin (top tertile) in conjunction with elevated NT-proBNP (≥125 pg/mL) (A+/N+) or nonelevated NT-proBNP (<125 pg/mL) (A+/N−) | 14–17 |
| **Supplemental Table 8:** | Top significant pathways associated with A+/N+ and A+/N− | 18 |
| **Supplemental Table 9:** | Associations of top 3 metabolites associated with A+/N+ and A+/N− with incident cardiovascular events | 19–20 |
| **Supplemental Table 10:** | "STROBE Statement—Checklist of items that should be included in reports of cohort studies" | 21-31 |
| **Supplemental Figure 1:** | Cohort derivation | 32 |
| **Supplemental Figure 2:** | Scatter plot of adiponectin and NT-proBNP levels | 33 |
| **Supplemental References** | | 34 |

## Supplemental Methods

*Study Population*

ARIC[1] is a prospective observational study of CVD incidence in adults aged 45–64 years when recruited from 4 U.S. communities in 1987–1989 (visit 1). The study protocol was approved by the institutional review boards of all participating centers, and all participants provided written informed consent. ARIC visit 5 (2011–2013) was the index visit for this analysis.

Of 6538 participants at visit 5, we excluded individuals with prevalent coronary heart disease (CHD; n=1013), stroke (n=174), or HF (n=444), race other than White or Black (n=18) and Black participants at the Minneapolis or Washington field centers (n=24) because of small numbers, and participants missing adiponectin data (n=136). Prevalent CHD and stroke were defined as self-reported MI or stroke before visit 1, ARIC-adjudicated MI or stroke, silent MI identified by ECG changes, or coronary revascularization between visits 1 and 5. [2] Prevalent HF was defined as signs and symptoms of HF by the Gothenburg criteria at visit 1 or HF hospitalization by diagnosis code (ICD-9 code 428) between visits 1 and 5. [3] After exclusions, 4729 participants were included for the primary analysis; metabolomic data were available for 4006 **(Supplemental Figure 1).**

All ARIC biospecimens were stored at a central biorepository, and biomarker measurements were performed in a core College of American Pathologists (CAP)–accredited/Clinical Laboratory Improvement Amendments (CLIA)–certified laboratory on previously unthawed ethylenediaminetetraacetic acid (EDTA) plasma samples. Effects of long-term storage on the reproducibility of various biomarkers in ARIC have been previously published.[4]

*Biomarker Assessment*

Adiponectin was measured (June 2017–December 2018) in EDTA plasma (collected 2011–2013 at visit 5; stored at −80°C and never thawed until immediately prior to laboratory testing) using an automated latex-enhanced immunoturbidimetric assay (Denka Seiken, Tokyo, Japan). The adiponectin assay had a limit of detection of 0.2 µg/mL with interassay coefficients of variation of 3.4% and 2.8% for control levels with mean adiponectin concentrations of 6.2 µg/mL and 13.1 µg/mL, respectively. The reliability coefficient was 0.98 based on 713 blinded quality control samples.

NT-proBNP was measured in EDTA plasma (collected at visit 5; stored at −70°C) using an electrochemiluminescent immunoassay on an automated Cobas e411 analyzer (Roche Diagnostics, Indianapolis, IN). The lower limit of detection for this assay is 5 pg/mL; the interassay coefficient of variance was 7.4% for a mean control level of 134 pg/mL. [5]

Cardiac structure and function were assessed by comprehensive 2-dimensional, Doppler, tissue Doppler, and speckle-tracking echocardiography performed at visit 5.[6] Parameters of interest for the present study included left ventricular ejection fraction (LVEF), left ventricular mass index (LVMI), average peak longitudinal strain, early diastolic mitral annular velocity (e′), transmitral early peak velocity to early diastolic mitral annular velocity ratio (E/e′), left atrial volume index (LAVI), and peak tricuspid regurgitation (TR) velocity. A predefined imaging protocol with uniform imaging hardware and software was used for echocardiographic acquisition and processing. Quantitative measures were according to American Society of Echocardiography guidelines.[7]

Metabolomic profiling was performed on visit 5 serum samples (stored at −80°C since collection) by Metabolon Inc (Durham, NC) using untargeted, gas chromatography/mass

spectrometry– and liquid chromatography/mass spectrometry–based quantification.[8] Identification of metabolites was performed as described previously. Analytes with medium-term reliability coefficients <0.60, based on a comparison of 2 samples collected 4–6 weeks apart from 60 individuals, were excluded from analysis as previously described.[8] Metabolite levels were winsorized at the 1st and 99th percentile (with missing/below-detection limit values given the lowest detected value of that metabolite) and standardized prior to analysis.

*Outcomes*

Primary outcomes were incident HF hospitalization, CHD, stroke, CVD mortality, and all-cause mortality. Incident HF hospitalization was ascertained by expert panel adjudication; HF was further adjudicated as HF with preserved ejection fraction (HFpEF: LVEF ≥ 50%) or HF with reduced ejection fraction (HFrEF: LVEF <50%).[9] Incident CHD events encompassed fatal CHD and definite or probable MI.[10] Incident stroke events included ischemic strokes and definite or probable hospitalized embolic or thrombotic strokes based on diagnosis codes as well as hospital records and neuroimaging reports.[11] CVD deaths were ascertained by diagnostic codes from hospital discharge records and from death certificates. The cut-off date for administrative censoring for those without events was December 31, 2017.

For exploratory outcomes, we used a validated physical frailty phenotype that encompassed measures of grip strength, walking speed, weight loss, energy, and physical activity to define robustness and frailty at visit 5. Individuals were classified as frail (≥3 components present), prefrail (1 or 2 components present), or robust (0 components present).[12]

*Statistical Analysis*

Adiponectin was modeled as both a continuous variable and a categorical variable (tertiles). For the continuous analysis, adiponectin values were log transformed. For the categorical analysis, the lowest tertile of adiponectin was used as the reference group. Cox proportional hazard models were constructed to assess associations between adiponectin at visit 5 and incident HF hospitalization, CHD, stroke, and CVD death after visit 5. The proportional hazard assumption was checked for all variables added to these models. Model 1 adjusted for age, sex, and race. Model 2 additionally adjusted for total cholesterol, high-density lipoprotein cholesterol (HDL-C), current smoking, systolic blood pressure, antihypertensive medication use, diabetes status, body mass index (BMI), estimated glomerular filtration rate (eGFR), lipid-lowering medication use, and high-sensitivity C-reactive protein (hs-CRP). Interaction was tested for sex, race, diabetes status, BMI (≥25 kg/$m^2$ vs <25 kg/$m^2$), and NT-proBNP (≥125 pg/mL vs <125 pg/mL); stratified analysis was performed for variables with significant interaction. Because of a high death rate after visit 5, we also performed sensitivity analysis evaluating risk for incident CVD events competing with nonevent death.[13] The shape of associations between adiponectin and outcome events was evaluated using Cox regression models adjusted for age, sex, and race with adiponectin levels modeled as restricted cubic splines; the median value was used as the reference with knots placed at the 5th, 27.5th, 50th, 72.5th, and 95th percentiles. Cross-sectional associations of adiponectin with NT-proBNP and echocardiographic parameters were investigated using linear regression analysis adjusting for covariables included in models 1 and 2 as described above.

To evaluate the relationship of adiponectin and NT-proBNP with CVD events further, we categorized participants based on adiponectin (using tertiles) and NT-proBNP levels (using

clinical cut-off of 125 pg/mL).[14] The four categories were elevated adiponectin (upper tertile) with elevated NT-proBNP (≥125 pg/mL) (A+/N+), nonelevated adiponectin (lower 2 tertiles) with elevated NT-proBNP (A−/N+), elevated adiponectin with nonelevated NT-proBNP (<125 pg/mL) (A+/N−), and nonelevated adiponectin with nonelevated NT-proBNP (A−/N−). Categories were compared with respect to risk for outcome events using Cox regression analysis with adjustment models 1 and 2 as above. The A+/N− group was used as the reference because this category had the lowest cardiometabolic risk profile (i.e., lower rates of hypertension, diabetes, and elevated BMI).

In exploratory analysis, we assessed the association of adiponectin and NT-proBNP categories with robustness and frailty evaluated at visit 5, using multinomial logistic regression adjusted for age, sex, and race.

For metabolomic analysis, logistic regression was used to compare elevated adiponectin (A+) with nonelevated adiponectin (A−) samples to identify metabolites associated with adiponectin level in the blood while adjusting for model 2 covariates as described above. Multivariable logistic regression including variables from model 2 was performed to identify metabolites with significantly different levels between A+/N+ and A+/N− participant subgroups. To correct for multiple testing, the resultant p-value was adjusted by using the Benjamini and Hochberg method to obtain the corresponding false discovery rate (FDR). Additionally, we performed least absolute shrinkage and selection operator logistic regression to identify a core set of metabolites that can distinguish A+/N+ versus A+/N− samples. The R package "glmnet" was implemented to carry out the analysis. Top metabolites of each subgroup were assessed for risk for outcome events using Cox regression analysis with adjustment model 2 as above.

Hosmer-Lemeshow goodness-of-fit test and Area Under the Receiver Operating Curves

were used to check the fitness of logistic regression models. Basic assumptions of linear regression models were followed: 1. Linearity, 2. Independence, 3. Homoscedasticity, 4. Normality, 5. Lack of multicollinearity. Linearity was checked by histogram and goodness-of-fit test. A non-linear transformation, such as log transformation, was applied if the data was not normally distributed. As the variations between the expected and observed values should be independent, scatterplots of residuals versus fits were applied to check the independence of errors. Homoscedasticity was tested by residual versus fitted plot. For every level of independent variables, the variance of errors remained constant. The normality of errors was checked by using Q-Q plots of the residuals and the Kolmogorov-Smirnov tests. Multicollinearity was tested by correlation matrix and variance inflation factor. Independent variables that showed high multicollinearity were excluded from the models.

Stata version 16 (StataCorp, College Station, TX), SAS version 9.4 (SAS Institute Inc., Cary, NC), and R version 3.3.3 were used for the statistical analyses. Orange was used for data visualization of metabolomics results.[15] Pathway analyses were performed using MetaboAnalyst 5.0.[16]

**Supplemental Table 1. Median adiponectin and NT-proBNP levels by sex**

| | Adiponectin (µg/mL) | NT-proBNP (pg/mL) |
|---|---|---|
| Male (N=1550) | 8.1 (5.2, 11.6) | 94.0 (50.6, 193.5) |
| Female (N=2456) | 12.1 (8.1, 17.6) | 137.7 (73.0, 239.6) |
| Total (N=4006) | 10.3 (6.8, 15.5) | 119.6 (63.2, 226.6) |

**Supplemental Table 2.** Associations of adiponectin (per in unit) with clinical characteristics, adjusted by age, sex, and race.

| | Unit increase | Beta coefficient | 95% CI | P value |
|---|---|---|---|---|
| **Age** | per 5-year | 0.09 | 0.08, 0.11 | <0.001 |
| **Female** | vs male | 0.43 | 0.40, 0.47 | <0.001 |
| **Black** | vs white | -0.40 | -0.44, -0.37 | <0.001 |
| **Total cholesterol** | per 10 mg/dL | 0.025 | 0.021, 0.029 | <0.001 |
| **HDL-C** | per 10 mg/dL | 0.20 | 0.19, 0.21 | <0.001 |
| **SBP** | per 10 mmHg | -0.004 | -0.014, 0.005 | 0.341 |
| **DBP** | per 10 mmHg | 0.005 | -0.011, 0.021 | 0.526 |
| **BP meds user** | vs non-user | -0.19 | -0.23, -0.16 | <0.001 |
| **Current smoker** | vs non-smoker | 0.07 | 0.004, 0.14 | 0.039 |
| **Diabetes** | vs non-diabetes | -0.29 | -0.33, -0.26 | <0.001 |
| **BMI** | per 5 $kg/m^2$ | -0.14 | -0.15, -0.12 | <0.001 |
| **eGFR** | per 10 mL/min/1.73 $m^2$ | 0.008 | -0.002, 0.018 | 0.131 |
| **Lipid lowering meds user** | vs non-user | -0.16 | -0.20, -0.13 | <0.001 |
| **ln-HS-CRP** | per ln unit | -0.09 | -0.10, -0.07 | <0.001 |
| **ln-NTproBNP** | per ln unit | 0.16 | 0.14, 0.18 | <0.001 |

**Supplemental Table 3.** Association of adiponectin and incident CVD events: competing risk analysis with nonevent death

| Event | Model | HR | 95% CI | P value |
|---|---|---|---|---|
| CHD (178/4729, 3.76%; nonevent death 525/4729, 11.10%) | 1 | 0.82 | 0.65-1.04 | 0.107 |
| | 2 | 1.14 | 0.83-1.56 | 0.427 |
| Ischemic stroke (115/4729, 2.43%; nonevent death 548/4729, 11.59%) | 1 | 0.65 | 0.50-0.85 | 0.002 |
| | 2 | 0.85 | 0.60-1.19 | 0.330 |
| HF hospitalization (325/4729, 6.87%; nonevent death 442/4729, 9.35%) | 1 | 1.30 | 1.05-1.62 | 0.017 |
| | 2 | 1.81 | 1.39-2.36 | <0.001 |
| HFpEF (220/4729, 4.65%; nonevent death 493/4729, 10.43%) | 1 | 1.38 | 1.05-1.80 | 0.019 |
| | 2 | 1.89 | 1.37-2.62 | <0.001 |
| HFrEF (131/4729, 2.77%; nonevent death 520/4729, 11.00%) | 1 | 1.09 | 0.80-1.49 | 0.584 |
| | 2 | 1.32 | 0.93-1.89 | 0.124 |

Adiponectin (natural log transformed) analyzed as a continuous variable. Model 1 adjusted by age, sex, and race; model 2 adjusted by model 1 covariables plus total cholesterol, HDL-C, current smoking, SBP, antihypertensive medication use, diabetes status, BMI, eGFR, lipid-lowering medication use, and log–hs-CRP.

**Supplemental Table 4.** Association of adiponectin tertile and incident CVD events

| Event | | Adiponectin tertiles (µg/mL) | | | P trend |
|---|---|---|---|---|---|
| | | 1 (0.2–7.8) | 2 (7.9–13.4) | 3 (13.5–50.0) | |
| Incident CHD | # events/at risk (%) | 71/1587 (4.47) | 63/1577 (3.99) | 44/1565 (2.81) | 0.042 |
| | incident rate in 1000 person-years | 8.43 (6.68, 10.64) | 7.60 (5.93, 9.72) | 5.47 (4.07, 7.35) | 0.0253 |
| | Model 1 | *Ref* | 0.92 (0.65-1.30) | 0.70 (0.47-1.06) | 0.2233 |
| | Model 2 | *Ref* | 1.14 (0.78-1.67) | 1.16 (0.72-1.88) | 0.7548 |
| Incident ischemic stroke | #events/at risk (%) | 56/1587 (3.53) | 31/1577 (1.97) | 28/1565 (1.79) | 0.002 |
| | incident rate in 1000 person-years | 6.63 (5.10, 8.61) | 3.70 (2.60, 5.26) | 3.46 (2.39, 5.01) | 0.0029 |
| | Model 1 | *Ref* | 0.57 (0.36-0.90) | 0.52 (0.32-0.85) | 0.0112 |
| | Model 2 | *Ref* | 0.63 (0.38-1.05) | 0.91 (0.51-1.61) | 0.1792 |
| Incident HF hospitalization | #events/at risk (%) | 104/1587 (6.55) | 93/1577 (5.90) | 128/1565 (8.18) | 0.034 |
| | incident rate in 1000 person-years | 12.37 (10.21, 14.99) | 11.22 (9.16, 13.75) | 16.15 (13.58, 19.20) | 0.0337 |
| | Model 1 | *Ref* | 0.99 (0.74-1.32) | 1.47 (1.10-1.96) | 0.0064 |
| | Model 2 | *Ref* | 1.15 (0.83-1.59) | 2.40 (1.71-3.38) | <0.0001 |
| Incident HFpEF | #events/at risk (%) | 70/1587 (4.41) | 60/1577 (3.80) | 90/1565 (5.75) | 0.030 |
| | incident rate in 1000 person-years | 8.27 (6.54, 10.45) | 7.21 (5.60, 9.28) | 11.25 (9.15, 13.83) | 0.0389 |
| | Model 1 | *Ref* | 0.93 (0.65-1.33) | 1.48 (1.04-2.09) | 0.0143 |
| | Model 2 | *Ref* | 1.14 (0.77-1.68) | 2.33 (1.55-3.52) | <0.0001 |
| Incident HFrEF | #events/at risk (%) | 51/1587 (3.21) | 35/1577 (2.22) | 45/1565 (2.88) | 0.223 |
| | incident rate in 1000 person-years | 6.00 (4.56, 7.89) | 4.17 (2.99, 5.81) | 5.59 (4.17, 7.48) | 0.7020 |
| | Model 1 | *Ref* | 0.77 (0.50- | 1.10 (0.71- | 0.2778 |

| | | | | | |
|---|---|---|---|---|---|
| | | | 1.20) | 1.71) | |
| | Model 2 | *Ref* | 0.84 (0.51-1.36) | 1.49 (0.89-2.49) | 0.0763 |
| CVD death | #events/at risk (%) | 62/1587 (3.91) | 44/1577 (2.79) | 74/1565 (4.73) | 0.017 |
| | incident rate in 1000 person-years | 7.23 (5.64, 9.27) | 5.21 (3.88, 7.01) | 9.09 (7.24, 11.42) | 0.1349 |
| | Model 1 | *Ref* | 0.81 (0.54-1.20) | 1.43 (0.98-2.08) | 0.0109 |
| | Model 2 | *Ref* | 0.90 (0.57-1.41) | 2.08 (1.32-3.29) | 0.0003 |

Model 1 adjusted by age, sex, and race; model 2 adjusted by model 1 covariables plus total cholesterol, HDL-C, current smoking, SBP, antihypertensive medication use, diabetes status, BMI, eGFR, lipid-lowering medication use, and log–hs-CRP.

**Supplemental Table 5.** Association of adiponectin and NT-proBNP categories with incident CVD

**A**. All subgroup analysis using A+/N- as Reference; **B**. A+/N+ vs A-/N+ (reference); **C**. A+/N- vs A-/N- (reference).

**A.**

| **Event** | | **Adiponectin <13.5 µg/mL, NT-proBNP <125 pg/mL** | **Adiponectin <13.5 µg/mL, NT-proBNP ≥125 pg/mL** | **Adiponectin ≥13.5 µg/mL, NT-proBNP <125 pg/mL** | **Adiponectin ≥13.5 µg/mL, NT-proBNP ≥125 pg/mL** |
|---|---|---|---|---|---|
| **Incident CHD** | #event/at risk (%) | 71/1945 (3.65) | 63/1219 (5.17) | 9/545 (1.65) | 35/1020 (3.43) |
| | Incident rate in 1000 person-years | 6.78 (5.38, 8.56) | 10.09 (7.88, 12.91) | 3.08 (1.60, 5.92) | 6.83 (4.91, 9.52) |
| | Model 1 | 1.88 (0.93-3.81) | 2.67 (1.32-5.41) | *Ref* | 2.01 (0.96-4.21) |
| | Model 2 | 1.34 (0.62-2.90) | 1.66 (0.76-3.62) | *Ref* | 1.87 (0.85-4.13) |
| **Incident ischemic stroke** | #event/at risk (%) | 46/1945 (2.37) | 41/1219 (3.36) | 5/545 (0.92) | 23/1020 (2.25) |
| | Incident rate in 1000 person-years | 4.37 (3.27, 5.83) | 6.52 (4.80, 8.85) | 1.71 (0.71, 4.10) | 4.45 (2.96, 6.70) |
| | Model 1 | 2.20 (0.86-5.62) | 3.06 (1.20-7.79) | *Ref* | 2.20 (0.83-5.83) |
| | Model 2 | 1.29 (0.49-3.40) | 1.67 (0.63-4.45) | *Ref* | 2.07 (0.77-5.55) |
| **Incident ASCVD** | #event/at risk (%) | 113/1945 (5.81) | 99/1219 (8.12) | 13/545 (2.39) | 56/1020 (5.49) |
| | Incident rate in 1000 person-years | 10.89 (9.06, 13.10) | 16.08 (13.20, 19.58) | 4.47 (2.59, 7.69) | 11.00 (8.46, 14.29) |

| | | | | | |
|---|---|---|---|---|---|
| | Model 1 | 2.13 (1.19-3.82) | 2.92 (1.63-5.22) | *Ref* | 2.15 (1.17-3.95) |
| | Model 2 | 1.43 (0.77-2.68) | 1.76 (0.94-3.31) | *Ref* | 2.03 (1.07-3.84) |
| **Incident HF hospitalization** | #event/at risk (%) | 65/1945 (3.34) | 132/1219 (10.83) | 10/545 (1.83) | 118/1020 (11.57) |
| | Incident rate in 1000 person-years | 6.18 (4.85, 7.88) | 21.38 (18.03, 25.36) | 3.41 (1.83, 6.34) | 23.64 (19.73, 28.31) |
| | Model 1 | 1.41 (0.72-2.75) | 4.77 (2.50-9.11) | *Ref* | 6.06 (3.17-11.60) |
| | Model 2 | 0.95 (0.46-1.96) | 2.84 (1.41-5.72) | *Ref* | 5.41 (2.72-10.78) |
| **HFpEF** | #event/at risk (%) | 57/1945 (2.93) | 73/1219 (5.99) | 9/545 (1.65) | 81/1020 (7.94) |
| | Incident rate in 1000 person-years | 5.41 (4.17, 7.02) | 11.66 (9.27, 14.67) | 3.08 (1.60, 5.92) | 15.95 (12.83, 19.83) |
| | Model 1 | 1.44 (0.71-2.95) | 3.04 (1.51-6.10) | *Ref* | 4.55 (2.28-9.11) |
| | Model 2 | 0.92 (0.44-1.91) | 1.75 (0.84-3.62) | *Ref* | 3.64 (1.80-7.36) |
| **HFrEF** | #event/at risk (%) | 20/1945 (1.03) | 66/1219 (5.41) | 1/545 (0.18) | 44/1020 (4.31) |
| | Incident rate in 1000 person-years | 1.88 (1.22, 2.92) | 10.50 (8.25, 13.37) | 0.34 (0.05, 2.41) | 8.61 (6.40, 11.56) |
| | Model 1 | 3.88 (0.52-29.03) | 21.77 (3.01-157.32) | *Ref* | 22.00 (3.02-160.04) |
| | Model 2 | 2.82 (0.37-21.38) | 14.25 (1.94-104.74) | *Ref* | 18.27 (2.49-133.77) |

| | | | | | |
|---|---|---|---|---|---|
| **CVD death** | #event/at risk (%) | 30/1945 (1.54) | 76/1219 (6.23) | 7/545 (1.28) | 67/1020 (6.57) |
| | Incident rate in 1000 person-years | 2.82 (1.97, 4.04) | 11.90 (9.51, 14.91) | 2.38 (1.13, 4.99) | 12.89 (10.15, 16.38) |
| | Model 1 | 0.86 (0.37-1.98) | 3.41 (1.57-7.44) | *Ref* | 4.27 (1.95-9.35) |
| | Model 2 | 0.56 (0.22-1.40) | 2.05 (0.86-4.89) | *Ref* | 3.50 (1.48-8.24) |

Model 1 adjusted by age, sex, and race; model 2 adjusted by model 1 covariables plus total cholesterol, HDL-C, current smoking, SBP, antihypertensive medication use, diabetes status, BMI, eGFR, lipid-lowering medication use, and log–hs-CRP.

**B.**

| | **A-/N+** | **A+/N+** | **P value** |
|---|---|---|---|
| **Incident ASCVD** | | | |
| #event/at risk (%) | 99/1219 (8.12) | 56/1020 (5.49) | 0.015 |
| Event rate (per 1000 person-year) | 16.08 (13.20, 19.58) | 11.00 (8.46, 14.29) | 0.022 |
| HR of Cox regression | Reference | 1.22 (0.82-1.81) | 0.331 |
| **Incident HF hospitalization** | | | |
| #event/at risk (%) | 132/1219 (10.83) | 118/1020 (11.57) | 0.580 |
| Event rate (per 1000 person-year) | 21.38 (18.03, 25.36) | 23.64 (19.73, 28.31) | 0.424 |
| HR of Cox regression | Reference | 1.82 (1.33-2.49) | <0.001 |
| **CVD death** | | | |
| #event/at risk (%) | 76/1219 (6.23) | 67/1020 (6.57) | 0.748 |
| Event rate (per 1000 person-year) | 11.90 (9.51, 14.91) | 12.89 (10.15, 16.38) | 0.583 |
| HR of Cox regression | Reference | 1.60 (1.05-2.45) | 0.030 |
| Model adjusted by age, sex, race, total cholesterol, HDL-C, current smoking, SBP, antihypertensive medication use, diabetes status, BMI, eGFR, lipid-lowering medication use, and log–hs-CRP. | | | |

**C.**

| | **A-/N-** | **A+/N-** | **P value** |
|---|---|---|---|
| **Incident ASCVD** | | | |
| #event/at risk (%) | 113/1945 (5.81) | 13/545 (2.39) | 0.001 |
| Event rate (per 1000 person-year) | 10.89 (9.06, 13.10) | 4.47 (2.59, 7.69) | 0.002 |
| HR of Cox regression | Reference | 0.58 (0.30-1.12) | 0.105 |

| | | | |
|---|---|---|---|
| **Incident HF hospitalization** | | | |
| #event/at risk (%) | 65/1945 (3.34) | 10/545 (1.83) | 0.069 |
| Event rate (per 1000 person-year) | 6.18 (4.85, 7.88) | 3.41 (1.83, 6.34) | 0.080 |
| HR of Cox regression | Reference | 1.20 (0.55-2.61) | 0.638 |
| **CVD death** | | | |
| #event/at risk (%) | 30/1945 (1.54) | 7/545 (1.28) | 0.660 |
| Event rate (per 1000 person-year) | 2.82 (1.97, 4.04) | 2.38 (1.13, 4.99) | 0.714 |
| HR of Cox regression | Reference | 1.91 (0.67-5.41) | 0.224 |
| Model adjusted by age, sex, race, total cholesterol, HDL-C, current smoking, SBP, antihypertensive medication use, diabetes status, BMI, eGFR, lipid-lowering medication use, and log–hs-CRP. | | | |

**Supplemental Table 6.** Association between adiponectin and NT-proBNP categories and robustness/frailty score by multinomial logistic regression model.

| | **Adiponectin <13.5 μg/mL, NT-proBNP <125 pg/mL** | **Adiponectin <13.5 μg/mL, NT-proBNP≥ 125 pg/mL** | **Adiponectin ≥13.5 μg/mL, NT-proBNP <125 pg/mL** | **Adiponectin ≥13.5 μg/mL, NT-proBNP ≥125 pg/mL** |
|---|---|---|---|---|
| **Model 1** | | | | |
| Prefrail vs frail | 0.99 (0.60-1.64) | 0.83 (0.50-1.36) | *Ref* | 0.67 (0.41-1.09) |
| Robust vs frail | 1.25 (0.75-2.07) | 0.76 (0.46-1.25) | *Ref* | 0.54 (0.33-0.89) |
| **Model 2** | | | | |
| Prefrail vs frail | 1.30 (0.76-2.24) | 1.10 (0.64-1.89) | *Ref* | 0.75 (0.44-1.25) |
| Robust vs frail | 2.11 (1.22-3.64) | 1.27 (0.73-2.21) | *Ref* | 0.59 (0.35-1.00) |

Data presented as odds ratio (95% CI). Model 1 adjusted by age, sex, and race; model 2 adjusted by model 1 covariables plus total cholesterol, HDL-C, current smoking, SBP, antihypertensive medication use, diabetes status, BMI, eGFR, lipid-lowering medication use, and log–hs-CRP.

**Supplemental Table 7.** Metabolites significantly associated with elevated adiponectin (top tertile) in conjunction with elevated NT-proBNP (≥125 pg/mL) (A+/N+) or nonelevated NT-proBNP (<125 pg/mL) (A+/N−)

| Metabolite | Superpathway | Subpathway | β | 95% CI | P value | FDR |
|---|---|---|---|---|---|---|
| **A+N+** | | | | | | |
| N2,N2-dimethylguanosine | nucleotide | purine metabolism, guanine containing | 0.51 | 0.31-0.72 | <0.001 | <0.001 |
| Hydroxyasparagine | amino acid | alanine and aspartate metabolism | 0.5 | 0.27-0.73 | <0.001 | 0.002 |
| 2,3-dihydroxy-5-methylthio-4-pentenoate | amino acid | methionine, cysteine, SAM, and taurine metabolism | 0.48 | 0.24-0.73 | <0.001 | 0.006 |
| 1-(1-enyl-palmitoyl)-2-oleoyl-GPC (P-16:0/18:1) | lipid | plasmalogen | 0.47 | 0.29-0.65 | <0.001 | <0.001 |
| Erythronate | carbohydrate | aminosugar metabolism | 0.39 | 0.15-0.63 | <0.001 | 0.031 |
| 1-oleoyl-GPC (18:1) | lipid | lysophospholipid | 0.38 | 0.21-0.56 | <0.001 | 0.002 |
| N-formylmethionine | amino acid | methionine, cysteine, SAM, and taurine metabolism | 0.37 | 0.19-0.56 | <0.001 | 0.004 |
| Malate | energy | TCA cycle | 0.37 | 0.22-0.53 | <0.001 | <0.001 |
| N6-carbamoylthreonyladenosine | nucleotide | purine metabolism, adenine containing | 0.36 | 0.18-0.55 | <0.001 | 0.006 |
| 5-methylthioribose | amino acid | methionine, cysteine, SAM, and taurine metabolism | 0.34 | 0.17-0.51 | <0.001 | 0.006 |
| 1-(1-enyl-palmitoyl)-2-palmitoleoyl-GPC (P-16:0/16:1) | lipid | plasmalogen | 0.34 | 0.16-0.52 | <0.001 | 0.011 |
| N-acetylserine | amino acid | glycine, serine, and threonine metabolism | 0.33 | 0.12-0.54 | 0.002 | 0.044 |
| Vanillactate | amino acid | tyrosine metabolism | 0.33 | 0.16-0.49 | <0.001 | 0.004 |

| | | | | | | |
|---|---|---|---|---|---|---|
| N-acetylneuraminate | carbohydrate | aminosugar metabolism | 0.32 | 0.14-0.5 | 0.001 | 0.019 |
| pseudouridine | nucleotide | pyrimidine metabolism, uracil containing | 0.31 | 0.1-0.53 | 0.004 | 0.048 |
| 1-stearoyl-GPE (18:0) | lipid | lysophospholipid | 0.31 | 0.16-0.47 | <0.001 | 0.004 |
| 1-ribosyl-imidazoleacetate | amino acid | histidine metabolism | 0.31 | 0.13-0.49 | 0.001 | 0.019 |
| 1,2-dipalmitoyl-GPC (16:0/16:0) | lipid | phosphatidylcholine | 0.31 | 0.17-0.45 | <0.001 | 0.002 |
| heptenedioate (C7:1-DC) | lipid | fatty acid, dicarboxylate | 0.3 | 0.14-0.46 | <0.001 | 0.007 |
| 4-hydroxyphenylacetylglutamine | peptide | acetylated peptides | 0.29 | 0.15-0.43 | <0.001 | 0.004 |
| sphingomyelin (d18:2/16:0, d18:1/16:1) | lipid | sphingomyelins | 0.29 | 0.11-0.47 | 0.001 | 0.031 |
| pyroglutamine | amino acid | glutamate metabolism | 0.29 | 0.11-0.46 | 0.002 | 0.033 |
| fumarate | energy | TCA cycle | 0.29 | 0.13-0.44 | <0.001 | 0.008 |
| 4-acetamidobutanoate | amino acid | polyamine metabolism | 0.28 | 0.11-0.45 | 0.001 | 0.026 |
| orotate | nucleotide | pyrimidine metabolism, orotate containing | 0.27 | 0.09-0.45 | 0.003 | 0.044 |
| N1-methylinosine | nucleotide | purine metabolism, (hypo)xanthine/inosine containing | 0.27 | 0.11-0.42 | 0.001 | 0.018 |
| 1-palmitoyl-2-stearoyl-GPC (16:0/18:0) | lipid | phosphatidylcholine | 0.26 | 0.13-0.4 | <0.001 | 0.007 |
| N-acetylaspartate | amino acid | alanine and aspartate metabolism | 0.26 | 0.11-0.41 | 0.001 | 0.017 |
| oleoylcholine | lipid | fatty acid metabolism (acyl choline) | 0.26 | 0.09-0.43 | 0.003 | 0.045 |
| 1,2-dilinoleoyl-GPC (18:2/18:2) | lipid | phosphatidylcholine | 0.25 | 0.09-0.41 | 0.003 | 0.044 |

| | | | | | | |
|---|---|---|---|---|---|---|
| 1-palmitoyl-2-linoleoyl-GPE (16:0/18:2) | lipid | phosphatidylethanolamine | 0.24 | 0.1-0.38 | 0.001 | 0.021 |
| 1-stearoyl-2-linoleoyl-GPI (18:0/18:2) | lipid | phosphatidylinositol | 0.24 | 0.09-0.39 | 0.001 | 0.031 |
| aconitate [cis or trans] | energy | TCA cycle | 0.24 | 0.08-0.4 | 0.003 | 0.045 |
| methionine sulfone | amino acid | methionine, cysteine, SAM, and taurine metabolism | 0.23 | 0.08-0.39 | 0.003 | 0.044 |
| 1-oleoyl-GPE (18:1) | lipid | lysophospholipid | 0.23 | 0.09-0.36 | 0.001 | 0.025 |
| 1-stearoyl-2-linoleoyl-GPE (18:0/18:2) | lipid | phosphatidylethanolamine | 0.23 | 0.09-0.37 | 0.002 | 0.034 |
| 3-amino-2-piperidone | amino acid | urea cycle; arginine and proline metabolism | 0.23 | 0.08-0.37 | 0.003 | 0.044 |
| 5-dodecenoylcarnitine (C12:1) | lipid | fatty acid metabolism (acyl carnitine, monounsaturated) | 0.22 | 0.07-0.37 | 0.004 | 0.05 |
| alpha-ketoglutarate | energy | TCA cycle | 0.22 | 0.08-0.35 | 0.002 | 0.038 |
| 1-palmitoyl-2-oleoyl-GPC (16:0/18:1) | lipid | phosphatidylcholine | 0.21 | 0.07-0.35 | 0.003 | 0.044 |
| 5,6-dihydrothymine | nucleotide | pyrimidine metabolism, thymine containing | 0.21 | 0.07-0.35 | 0.003 | 0.044 |
| 1-palmitoyl-2-arachidonoyl-GPE (16:0/20:4) | lipid | phosphatidylethanolamine | 0.21 | 0.07-0.35 | 0.003 | 0.044 |
| decadienedioic acid (C10:2-DC) | lipid | fatty acid, dicarboxylate | 0.21 | 0.07-0.35 | 0.003 | 0.045 |
| carboxyethyl-GABA | amino acid | glutamate metabolism | 0.21 | 0.07-0.35 | 0.003 | 0.045 |
| 1-stearoyl-2-arachidonoyl-GPE (18:0/20:4) | lipid | phosphatidylethanolamine | 0.21 | 0.07-0.35 | 0.004 | 0.048 |
| 1-stearoyl-2-oleoyl-GPC (18:0/18:1) | lipid | phosphatidylcholine | 0.2 | 0.07-0.33 | 0.003 | 0.044 |
| succinate | energy | TCA cycle | 0.2 | 0.06-0.34 | 0.004 | 0.048 |

| | | | | | | |
|---|---|---|---|---|---|---|
| 1-oleoyl-GPI (18:1) | lipid | lysophospholipid | 0.2 | 0.06-0.34 | 0.004 | 0.048 |
| dihomo-linoleoylcarnitine (C20:2) | lipid | fatty acid metabolism (acyl carnitine, polyunsaturated) | 0.2 | 0.06-0.33 | 0.004 | 0.048 |
| homovanillate | amino acid | tyrosine metabolism | 0.2 | 0.07-0.33 | 0.003 | 0.045 |
| hexadecenedioate (C16:1-DC) | lipid | fatty acid, dicarboxylate | 0.2 | 0.06-0.33 | 0.004 | 0.050 |
| 1-palmitoyl-2-oleoyl-GPE (16:0/18:1) | lipid | phosphatidylethanolamine | 0.19 | 0.06-0.31 | 0.004 | 0.048 |
| **A+N−** | | | | | | |
| leucine | amino acid | leucine, isoleucine, and valine metabolism | 0.41 | 0.24-0.58 | <0.001 | 0.001 |
| creatine | amino acid | creatine metabolism | 0.37 | 0.21-0.53 | <0.001 | 0.001 |
| isoleucine | amino acid | leucine, isoleucine, and valine metabolism | 0.36 | 0.2-0.53 | <0.001 | 0.002 |
| salicylate | xenobiotics | drug—topical agents | 0.28 | 0.14-0.41 | <0.001 | 0.004 |
| metabolonic lactone sulfate | partially characterized molecules | partially characterized molecules | 0.27 | 0.12-0.42 | <0.001 | 0.012 |
| threonylphenylalanine | peptide | dipeptide | 0.25 | 0.09-0.41 | 0.002 | 0.038 |
| arginine | amino acid | urea cycle; arginine and proline metabolism | 0.25 | 0.1-0.4 | 0.001 | 0.028 |
| 2-hydroxyhippurate (salicylurate) | xenobiotics | benzoate metabolism | 0.24 | 0.11-0.38 | <0.001 | 0.015 |
| 3-methyl-2-oxovalerate | amino acid | leucine, isoleucine and valine metabolism | 0.24 | 0.08-0.39 | 0.003 | 0.044 |
| homoarginine | amino acid | urea cycle; arginine and proline metabolism | 0.24 | 0.09-0.38 | 0.001 | 0.031 |
| methionine | amino acid | methionine, cysteine, SAM, and taurine metabolism | 0.23 | 0.09-0.38 | 0.002 | 0.033 |

| | | | | | | |
|---|---|---|---|---|---|---|
| 2-oxoarginine | amino acid | urea cycle; arginine and proline metabolism | 0.23 | 0.08-0.38 | 0.002 | 0.041 |
| fibrinopeptide A (2-15) | peptide | fibrinogen cleavage peptide | 0.22 | 0.07-0.38 | 0.004 | 0.048 |
| nisinate (24:6n3) | lipid | long-chain polyunsaturated fatty acid (n3 and n6) | 0.21 | 0.08-0.34 | 0.002 | 0.033 |
| fibrinopeptide A (3-15) | peptide | fibrinogen cleavage peptide | 0.21 | 0.07-0.35 | 0.004 | 0.048 |
| androsterone sulfate | lipid | androgenic steroids | 0.2 | 0.06-0.33 | 0.004 | 0.048 |
| 1-stearoyl-2-docosahexaenoyl-GPC (18:0/22:6) | lipid | phosphatidylcholine | 0.19 | 0.07-0.32 | 0.003 | 0.044 |

**Supplemental Table 8.** Top significant pathways associated with A+/N+ and A+/N−

| Pathway | Total | Expected | Hits | Raw P value | Holm-adjusted P value | FDR | Impact |
|---|---|---|---|---|---|---|---|
| **A+N+** | | | | | | | |
| Citrate cycle (TCA cycle) | 20 | 0.15484 | 4 | <0.001 | <0.001 | <0.001 | 0.17 |
| Alanine, aspartate, and glutamate metabolism | 28 | 0.21677 | 4 | <0.001 | <0.001 | 0.002 | 0.14 |
| **A+/N−** | | | | | | | |
| Aminoacyl-tRNA biosynthesis | 48 | 0.21677 | 4 | <0.001 | 0.002 | 0.002 | 0 |
| Valine, leucine, and isoleucine biosynthesis | 8 | 0.036129 | 2 | <0.001 | 0.040 | 0.020 | 0 |

A+/N+ = adiponectin in the top tertile and NT-proBNP ≥125 pg/mL
A+/N− = adiponectin in the top tertile and NT-proBNP <125 pg/mL

**Supplemental Table 9.** Associations of top 3 metabolites associated with A+/N+ and A+/N− with incident cardiovascular events.

| **A+/N+** | | | | | | |
|---|---|---|---|---|---|---|
| **Incident event (n/N, %)** | **N2,N2-dimethylguanosine** | | **Hydroxyasparagine** | | **DMPTA** | |
| | **HR (95% CI)** | **P value** | **HR (95% CI)** | **P value** | **HR (95% CI)** | **P value** |
| CHD (240/4006, 5.99%) | 1.06 (0.86-1.31) | 0.569 | 1.16 (0.92-1.46) | 0.219 | 1 (0.79-1.26) | 0.99 |
| Ischemic stroke (156/4006, 3.89%) | 1.33 (1.01-1.74) | **0.041** | 1.04 (0.77-1.40) | 0.801 | 1.26 (0.94-1.69) | 0.126 |
| HF hospitalization (487/4006, 12.16%) | 1.65 (1.41-1.93) | **<0.001** | 1.58 (1.34-1.87) | **<0.001** | 1.41 (1.19-1.67) | **<0.001** |
| HFpEF (336/4006, 8.39%) | 1.59 (1.31-1.92) | **<0.001** | 1.54 (1.26-1.89) | **<0.001** | 1.4 (1.15-1.71) | **0.001** |
| HFrEF (196/4006, 4.89%) | 1.47 (1.16-1.86) | **0.002** | 1.28 (0.99-1.66) | **0.059** | 1.08 (0.84-1.39) | 0.561 |
| CVD death (336/4006, 8.39%) | 2.1 (1.73-2.55) | **<0.001** | 1.72 (1.40-2.11) | **<0.001** | 1.54 (1.26-1.89) | **<0.001** |
| Total mortality (989/4006, 24.69%) | 1.62 (1.45-1.82) | **<0.001** | 1.53 (1.36-1.72) | **<0.001** | 1.42 (1.26-1.60) | **<0.001** |
| **A+/N−** | | | | | | |
| | **Leucine** | | **Creatine** | | **Isoleucine** | |
| **Incident event** | **HR (95% CI)** | **P value** | **HR (95% CI)** | **P value** | **HR (95% CI)** | **P value** |
| CHD (240/4006, 5.99%) | 1 (0.85-1.17) | 0.96 | 0.93 (0.80-1.08) | 0.34 | 1 (0.85-1.17) | 0.993 |

| | | | | | | |
|---|---|---|---|---|---|---|
| Ischemic stroke (156/4006, 3.89%) | 1.1 (0.90-1.35) | 0.343 | 1.04 (0.85-1.27) | 0.684 | 1.16 (0.95-1.42) | 0.146 |
| HF hospitalization (487/4006, 12.16%) | 0.8 (0.73-0.88) | **<0.001** | 0.94 (0.84-1.05) | 0.246 | 0.86 (0.77-0.96) | **0.01** |
| HFpEF (336/4006, 8.39%) | 0.78 (0.70-0.88) | **<0.001** | 0.9 (0.79-1.03) | 0.143 | 0.85 (0.74-0.97) | **0.017** |
| HFrEF (196/4006, 4.89%) | 0.77 (0.67-0.89) | **<0.001** | 1.04 (0.88-1.24) | 0.622 | 0.78 (0.66-0.93) | **0.004** |
| CVD death (336/4006, 8.39%) | 0.8 (0.71-0.90) | **<0.001** | 0.85 (0.75-0.97) | **0.016** | 0.93 (0.81-1.07) | 0.319 |
| Total mortality (989/4006, 24.69%) | 0.83 (0.77-0.89) | **<0.001** | 0.87 (0.81-0.94) | **0.001** | 0.96 (0.89-1.04) | 0.338 |

Follow-up years from the date of visit 5 to December 31, 2020. Mean follow-up is 7.7±2.01 years; median follow-up is 8.42 (7.62, 8.94) years. Model adjusted by age, sex, race, total cholesterol, HDL-C, current smoking, SBP, antihypertensive medication use, diabetes status, BMI, eGFR, lipid-lowering medication use, and log–hs-CRP. A+/N+ = adiponectin in the top tertile and NT-proBNP ≥125 pg/mL; A+/N− = adiponectin in the top tertile and NT-proBNP <125 pg/mL; CHD=coronary heart disease, HF=heart failure, HFpEF=heart failure with preserved ejection fraction, HFrEF= heart failure with reduced ejection fraction, CVD=cardiovascular disease, DMPTA=2,3-dihydroxy-5-methylthio-4-pentenoate.

**Supplemental Figure 10:** "STROBE Statement—Checklist of items that should be included in reports of ***cohort studies***"[17]

| | Item No | Recommendation | Page Number | Relevant Section |
|---|---|---|---|---|
| **Title and abstract** | 1 | (*a*) Indicate the study's design with a commonly used term in the title or the abstract | 3 | Abstract Methods |
| | | (*b*) Provide in the abstract an informative and balanced summary of what was done and what was found | 3 | Abstract Methods and Results |
| **Introduction** | | | | |
| Background/rationale | 2 | Explain the scientific background and rationale for the investigation being reported | 5 | Introduction |
| Objectives | 3 | State specific objectives, including any prespecified hypotheses | 6 | "We hypothesized that NTproBNP impacts the association of adiponectin with HF." |
| **Methods** | | | | |
| Study design | 4 | Present key elements of study design early in the paper | 6 | Methods: "ARIC[17] is a prospective observational cohort study of CVD incidence in adults recruited from 4 U.S. communities." |
| Setting | 5 | Describe the setting, locations, and relevant dates, including periods of recruitment, exposure, follow-up, and data collection | 6-7 and Supplement | Methods: "ARIC visit 5 (2011–2013) was the index visit for this analysis."…" The cut-off date for administrative censoring for events was December 31, 2017, for the |

| | | | | |
|---|---|---|---|---|
| | | | | primary analysis and December 31, 2020, for metabolic profiling."<br><br>Supplemental Methods: "ARIC[1] is a prospective observational study of CVD incidence in adults aged 45–64 years when recruited from 4 U.S. communities in 1987–1989 (visit 1). The study protocol was approved by the institutional review boards of all participating centers, and all participants provided written informed consent. ARIC visit 5 (2011–2013) was the index visit for this analysis."<br><br>Information on the ARIC study has been previously published in detail and thus is referenced in manuscript (reference 17) and supplemental material (reference 1). |
| Participants | 6 | (*a*) Give the eligibility criteria, and the sources and methods of selection of participants. Describe methods of follow-up | 9 | Results, Paragraph 1 |
| | | (*b*) For matched studies, give matching criteria and number of exposed and unexposed | N/A | N/A |

| | | | | |
|---|---|---|---|---|
| Variables | 7 | Clearly define all outcomes, exposures, predictors, potential confounders, and effect modifiers. Give diagnostic criteria, if applicable | 7 | Methods, "Outcomes" section |
| Data sources/ measurement | 8* | For each variable of interest, give sources of data and details of methods of assessment (measurement). Describe comparability of assessment methods if there is more than one group | 6-7 | Methods, "Biomarkers" section |
| Bias | 9 | Describe any efforts to address potential sources of bias | 8 | Methods, "Statistical Analysis" |
| Study size | 10 | Explain how the study size was arrived at | 9 | Results, Paragraph 1 |
| Quantitative variables | 11 | Explain how quantitative variables were handled in the analyses. If applicable, describe which groupings were chosen and why | 7<br><br>8 | "Adiponectin was evaluated in categorical (tertiles) and continuous (natural log–transformed values) analyses."<br><br>"Shapes of associations between adiponectin and outcomes were evaluated with adiponectin levels modeled as restricted cubic splines." |
| Statistical methods | 12 | (*a*) Describe all statistical methods, including those used to control for confounding | 7-8 | Methods, "Statistical Analysis" section |

| | | |
|---|---|---|
| (*b*) Describe any methods used to examine subgroups and interactions | 8 | "Interaction was tested for sex, race, diabetes status, BMI, and NT-proBNP; stratified analysis was performed for variables with significant interaction."<br><br>"The relationship of adiponectin and NT-proBNP with CVD events was further evaluated by categorizing participants by both adiponectin and NT-proBNP levels: elevated adiponectin (upper tertile) with elevated NT-proBNP (≥125 pg/mL) (A+/N+), nonelevated adiponectin (lower 2 tertiles) with elevated NT-proBNP (A−/N+), elevated adiponectin with nonelevated NT-proBNP (<125 pg/mL) (A+/N−), and nonelevated adiponectin with nonelevated NT-proBNP (A−/N−). Risk for outcome events was compared across categories using Cox regression models described above. The A+/N− subgroup was used as reference because it had the lowest cardiometabolic risk profile (lower rates of hypertension, diabetes, and elevated BMI)." |

| | | | | |
|---|---|---|---|---|
| | | (*c*) Explain how missing data were addressed | 9 | Restuls, Paragraph 1 |
| | | (*d*) If applicable, explain how loss to follow-up was addressed | N/A | N/A |
| | | (*e*) Describe any sensitivity analyses | 8 | "Because of a high death rate after visit 5, we performed sensitivity analysis evaluating risk for incident CVD events competing with nonevent death." |
| **Results** | | | | |
| Participants | 13* | (a) Report numbers of individuals at each stage of study—eg numbers potentially eligible, examined for eligibility, confirmed eligible, included in the study, completing follow-up, and analysed | 9 | Results, Paragraph 1 |
| | | (b) Give reasons for non-participation at each stage | 9 | Results, Paragraph 1 |
| | | (c) Consider use of a flow diagram | Supplemental | Supplemental Figure 1 |
| Descriptive data | 14* | (a) Give characteristics of study participants (eg demographic, clinical, social) and information on exposures and potential confounders | 9 | Table 1 |
| | | (b) Indicate number of participants with missing data for each variable of interest | N/A | N/A |

| | | | | |
|---|---|---|---|---|
| | | (c) Summarise follow-up time (eg, average and total amount) | 10 | "Over a median follow-up of 5.5 years (5.09, 6.03),…" |
| Outcome data | 15* | Report numbers of outcome events or summary measures over time | 11 | Table 3 |
| Main results | 16 | (*a*) Give unadjusted estimates and, if applicable, confounder-adjusted estimates and their precision (eg, 95% confidence interval). Make clear which confounders were adjusted for and why they were included | 11, Supplemental | Table 3<br>Supplemental Table 2 |
| | | (*b*) Report category boundaries when continuous variables were categorized | 9 | Table 1 |
| | | (*c*) If relevant, consider translating estimates of relative risk into absolute risk for a meaningful time period | Supplemental | Supplemental Table 5 |
| Other analyses | 17 | Report other analyses done—eg analyses of subgroups and interactions, and sensitivity analyses | 12 | Results, "Adiponectin, NT-proBNP and CVD Risk" section |
| **Discussion** | | | | |
| Key results | 18 | Summarise key results with reference to study objectives | 14 | Discussion, Paragraph 1 |
| Limitations | 19 | Discuss limitations of the study, taking into account sources of potential bias or | 17 | Discussion, Paragraph 7, "Our study has several limitations…" |

| | | | | |
|---|---|---|---|---|
| | | imprecision. Discuss both direction and magnitude of any potential bias | | |
| Interpretation | 20 | Give a cautious overall interpretation of results considering objectives, limitations, multiplicity of analyses, results from similar studies, and other relevant evidence | 17 | Discussion, Paragraph 6, “In summary, …” |
| Generalisability | 21 | Discuss the generalisability (external validity) of the study results | 18 | “Overall, the extensive data available from the ARIC study, including metabolomic data, enabled a more comprehensive perspective on the association between adiponectin and cardiovascular health in older adults and facilitated the identification of a high-risk phenotype characterized by elevated adiponectin and NT-proBNP.” |
| **Other information** | | | | |
| Funding | 22 | Give the source of funding and the role of the funders for the present study and, if applicable, for the original study on which the present article is based | 1 | Title page—“Sources of Funding |

**Supplemental Figure 1.** Cohort derivation

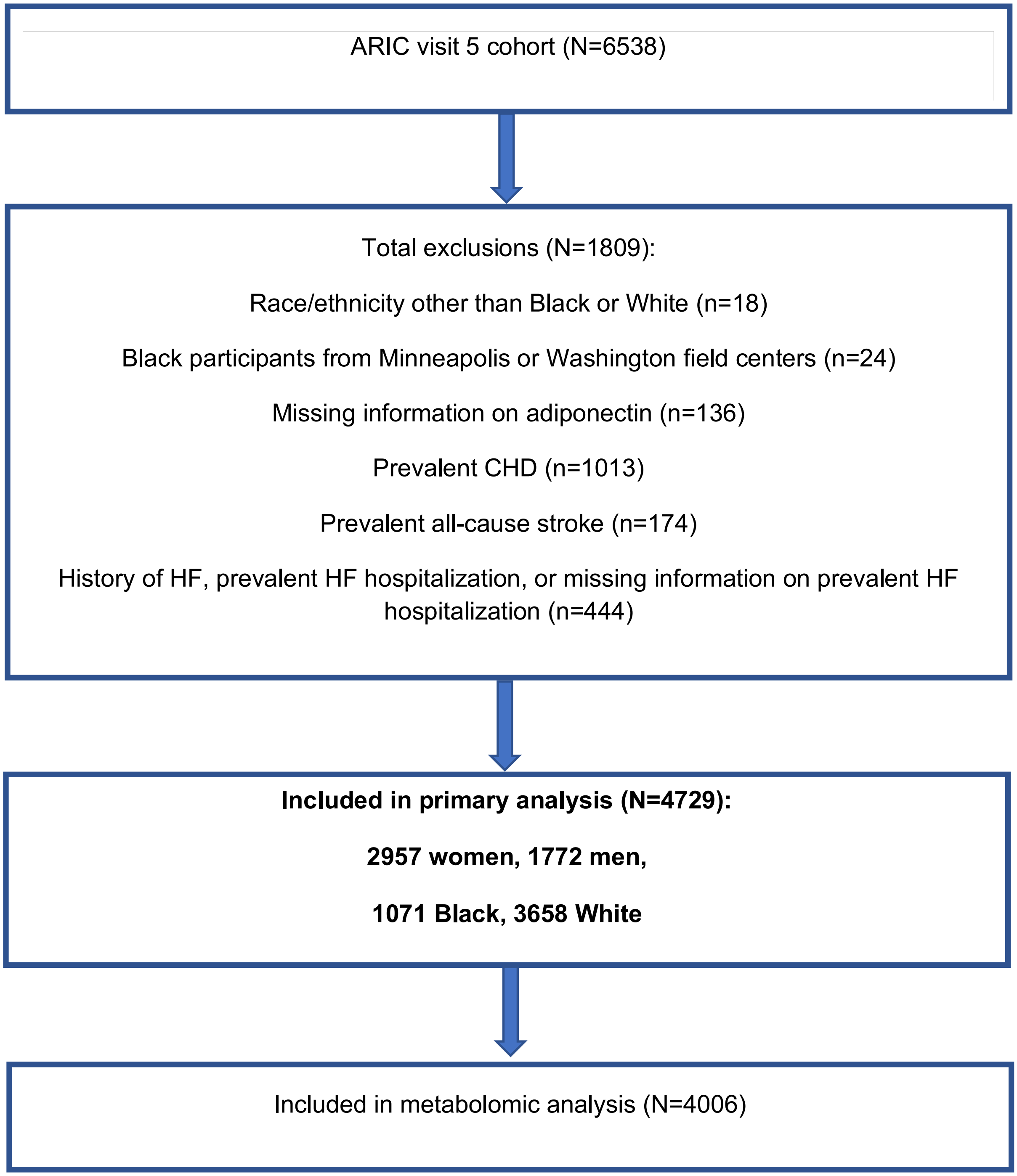

**Supplemental Figure 2. Scatter plot of adiponectin and NT-proBNP levels.** Red lines represent upper tertile cutpoint for adiponectin at 13.5 µg/mL (vertical) and NT-proBNP of 125 pg/mL (horizontal).

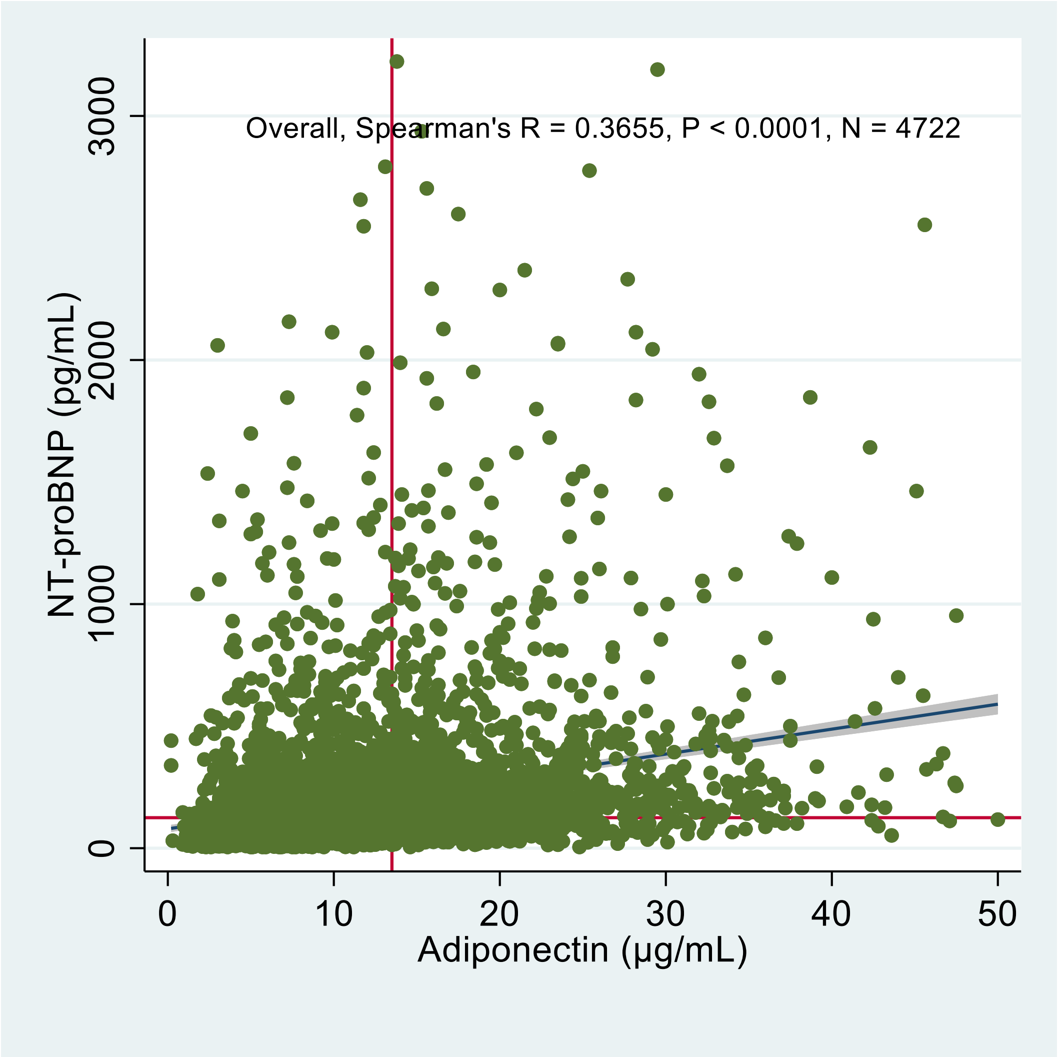

**Supplemental References**

1. Wright JD, Folsom AR, Coresh J, et al. The ARIC (Atherosclerosis Risk In Communities) Study: JACC Focus Seminar 3/8. *J Am Coll Cardiol*. Jun 15 2021;77(23):2939-2959. doi:10.1016/j.jacc.2021.04.035
2. Saunders JT, Nambi V, de Lemos JA, et al. Cardiac troponin T measured by a highly sensitive assay predicts coronary heart disease, heart failure, and mortality in the Atherosclerosis Risk in Communities Study. *Circulation*. Apr 5 2011;123(13):1367-76. doi:10.1161/CIRCULATIONAHA.110.005264
3. Loehr LR, Rosamond WD, Chang PP, Folsom AR, Chambless LE. Heart failure incidence and survival (from the Atherosclerosis Risk in Communities study). *Am J Cardiol*. Apr 1 2008;101(7):1016-22. doi:10.1016/j.amjcard.2007.11.061
4. Parrinello CM, Grams ME, Couper D, et al. Recalibration of blood analytes over 25 years in the atherosclerosis risk in communities study: impact of recalibration on chronic kidney disease prevalence and incidence. *Clin Chem*. Jul 2015;61(7):938-47. doi:10.1373/clinchem.2015.238873
5. Madan N, Lee AK, Matsushita K, et al. Relation of Isolated Systolic Hypertension and Pulse Pressure to High-Sensitivity Cardiac Troponin-T and N-Terminal pro-B-Type Natriuretic Peptide in Older Adults (from the Atherosclerosis Risk in Communities Study). *Am J Cardiol*. Jul 15 2019;124(2):245-252. doi:10.1016/j.amjcard.2019.04.030
6. Shah AM, Cheng S, Skali H, et al. Rationale and design of a multicenter echocardiographic study to assess the relationship between cardiac structure and function and heart failure risk in a biracial cohort of community-dwelling elderly persons: the Atherosclerosis Risk in Communities study. *Circ Cardiovasc Imaging*. Jan 2014;7(1):173-81. doi:10.1161/CIRCIMAGING.113.000736
7. Lang RM, Badano LP, Mor-Avi V, et al. Recommendations for cardiac chamber quantification by echocardiography in adults: an update from the American Society of Echocardiography and the European Association of Cardiovascular Imaging. *Eur Heart J Cardiovasc Imaging*. Mar 2015;16(3):233-70. doi:10.1093/ehjci/jev014
8. Zheng Y, Yu B, Alexander D, et al. Associations between metabolomic compounds and incident heart failure among African Americans: the ARIC Study. *Am J Epidemiol*. Aug 15 2013;178(4):534-42. doi:10.1093/aje/kwt004
9. Rosamond WD, Chang PP, Baggett C, et al. Classification of heart failure in the atherosclerosis risk in communities (ARIC) study: a comparison of diagnostic criteria. *Circ Heart Fail*. Mar 1 2012;5(2):152-9. doi:10.1161/CIRCHEARTFAILURE.111.963199
10. Rosamond WD, Chambless LE, Folsom AR, et al. Trends in the incidence of myocardial infarction and in mortality due to coronary heart disease, 1987 to 1994. *N Engl J Med*. Sep 24 1998;339(13):861-7. doi:10.1056/NEJM199809243391301
11. Rosamond WD, Folsom AR, Chambless LE, et al. Stroke incidence and survival among middle-aged adults: 9-year follow-up of the Atherosclerosis Risk in Communities (ARIC) cohort. *Stroke*. Apr 1999;30(4):736-43.
12. Kucharska-Newton AM, Palta P, Burgard S, et al. Operationalizing Frailty in the Atherosclerosis Risk in Communities Study Cohort. *J Gerontol A Biol Sci Med Sci*. Mar 1 2017;72(3):382-388. doi:10.1093/gerona/glw144
13. Fine JP, Gray RJ. A Proportional Hazards Model for the Subdistribution of a Competing Risk. *Journal of the American Statistical Association*. 1999;94(446):496-509.

14. Mueller C, McDonald K, de Boer RA, et al. Heart Failure Association of the European Society of Cardiology practical guidance on the use of natriuretic peptide concentrations. *Eur J Heart Fail*. Jun 2019;21(6):715-731. doi:10.1002/ejhf.1494

15. Demšar J, Curk T, Erjavec A, et al. Orange: data mining toolbox in Python. *J Mach Learn Res*. 2013;14(35):2349-2353.

16. McGill University, Xia Lab. MetaboAnalyst 5.0. Xia Lab @ McGill (last updated 2023-07-25). Updated 2023 Jul 25. Accessed 27 July 2023, https://www.metaboanalyst.ca/

17. von Elm E, Altman DG, Egger M, et al. The Strengthening the Reporting of Observational Studies in Epidemiology (STROBE) statement: guidelines for reporting observational studies. *J Clin Epidemiol*. Apr 2008;61(4):344-9. doi:10.1016/j.jclinepi.2007.11.008
